# N-terminal pyroglutamylation of an HLA-A24-restricted immunodominant epitope enhances SARS-CoV-2-specific T-cell responses

**DOI:** 10.3389/fimmu.2026.1758642

**Published:** 2026-04-27

**Authors:** Yoshiki Aritsu, Masahiro Kitabatake, Amiri Kurose, Hiroshi Hamana, Takeshi Nakama, Kyoko Yamada, Ryutaro Furukawa, Noriko Ouji-Sageshima, Atsushi Hara, Kaito Yasuike, Suzu Itami, Huanyu Li, Keiko Udaka, Hiroyuki Kishi, Takamasa Ueno, So Nakagawa, Toshihiro Ito, Norihito Kawashita, Mizuki Kitamatsu, Chihiro Motozono

**Affiliations:** 1 Division of Infection and Immunity, Joint Research Center for Human Retrovirus Infection, Kumamoto University, Kumamoto, Japan; 2 Department of Immunology, Nara Medical University, Nara, Japan; 3 Department of Applied Chemistry, Faculty of Science and Engineering, Kindai University, Osaka, Japan; 4 Department of Immunology, Faculty of Medicine, Academic Assembly, University of Toyama, Toyama, Japan; 5 Graduate School of Science and Engineering, Kindai University, Osaka, Japan; 6 Department of Immunology, Kochi University, Kochi, Japan; 7 Department of Molecular Life Science, Tokai University School of Medicine, Kanagawa, Japan; 8 Department of Energy and Materials, Faculty of Science and Engineering, Kindai University, Osaka, Japan

**Keywords:** peptide vaccine, pyroglutamate, SARS-CoV-2, T cell, TCR (T cell receptor)

## Abstract

T-cell responses play a critical role in the control of SARS-CoV-2 infections. Here, we examined the T-cell response to a conserved, immunodominant HLA-A*24:02-restricted SARS-CoV-2 spike protein epitope, QI9/A24 (QYIKWPWYI; residues 1208–1216). TCR repertoire analysis revealed that QI9/A24-specific T cells are highly diverse, with 219 clonotypes isolated and 169 αβ TCR pairs identified in six donors. To further characterize this response, we evaluated T-cell recognition of alanine-substituted and homologous coronavirus-derived peptides, using a TCR reconstitution system, and found that QI9/A24 TCRs tolerated numerous substitutions at the N-terminus and position 3. During peptide synthesis, we unexpectedly discovered that an N-terminally pyroglutamylated QI9/A24 peptide (pyrQI9) exhibited increased protease resistance, compared with the unmodified peptide. Further analysis revealed that the pyrQI9 peptide was recognized with higher sensitivity than the wild-type sequence and efficiently induced antigen-specific T-cell responses at low concentrations in both vaccinated and convalescent HLA-A*24:02-positive individuals. Moreover, immunization of HLA-A24 transgenic mice with the pyrQI9 peptide elicited antigen-specific T-cell responses more efficiently than the wild-type peptide and conferred superior T-cell–mediated protection. Together, our findings suggest that subtle modifications of antigenic peptides at a tolerable site in the N-terminus with pyroglutamate can enhance T-cell immunity in SARS-CoV-2 infection, providing critical insights into the design of next-generation T-cell-based vaccines against viral infections.

## Introduction

T-cell receptors (TCRs) on cytotoxic CD8^+^ T lymphocytes (CTLs) recognize complexes formed by short peptide fragments bound to HLA class I molecules. These peptides are typically generated by proteasomes from cytosolic proteins within target cells and then transported into the lumen of the endoplasmic reticulum (ER) by the transporter associated with antigen presentation (TAP), where they are loaded onto HLA class I molecules (1). The peptide-HLA (pHLA) complexes are then translocated to the cell surface for recognition by TCRs on CD8^+^ CTLs.

The T-cell response plays a crucial role in controlling viral infections. Early studies indicated that the size/nature of T-cell responses specific for SARS-CoV-2 correlate with COVID-19 severity (2, 3). Additionally, a recent study showed that HLA-B*15:01 is linked to asymptomatic SARS-CoV-2 infection, highlighting the critical role of HLA-B*15:01-restricted T cells in combating virally infected cells (4). This strong and dominant SARS-CoV-2-specific T-cell response can be enhanced by preexisting cross-reactive T cells elicited during prior coronavirus infections (4–6).

The HLA-A*24:02 allele is one of the most frequent and widely distributed HLA-I alleles worldwide (7, 8) and is present in ~60% of the Japanese cohort we studied (9). During the COVID-19 pandemic, there was an inverse correlation between SARS-CoV-2-related deaths per 100,000 people and the proportion of the population harboring the HLA-A*24:02 allele (10). We and others previously identified two immunodominant epitopes from the SARS-CoV-2 spike (S) protein, NF9/A24 (residues 448-456; NYNYLYRLF) and QI9/A24 (residues 1208–1216; QYIKWPWYI) in HLA-A*24:02^+^-vaccinated donors and convalescents (8, 9, 11–15). We previously demonstrated that the NF9/A24 epitope with L452R and L452Q mutations from Delta/Epsilon and Lambda variants, respectively, conferred escape from NF9/A24-specific T-cell responses in the context of HLA-A*24:02 (8, 16). The T-cell immune escape was also observed in HLA-A*24:02^+^ convalescents infected with Omicron BA.2.86 and JN.1 variants (14). On the other hand, the QI9/A24 epitope is conserved across SARS-CoV-2 variants (9, 12, 15). Moreover, we previously demonstrated that QI9/A24-specific CD8^+^ T cells comparably suppressed viral replication of prototype, Delta, and Omicron subvariants (9). The SARS-CoV-2-derived QI9/A24 peptide sequence showed high homology with other coronaviruses, such as SARS-CoV-1, MERS, and common cold coronaviruses (12, 15), indicating the possibility of preexisting CD8^+^ T cells before infection, such as HLA-B*15:01-restricted spike-specific CD8^+^ T cells associated with asymptomatic SARS-CoV-2 infection (4). These data suggest that CD8^+^ T cells specific for the conserved immunodominant epitope, such as the QI9/A24 in the context of HLA-A*24:02, may play an important role in combating virally infected cells across SARS-CoV-2 variants.

In this study, we characterized the clonotypes and cross-reactive profiles of TCRs specific to the QI9/A24 peptide. Moreover, during the QI9/A24 peptide synthesis, we identified the N-terminally-pyroglutamate QI9 peptide (pyrQI9) that effectively induces QI9/A24-specific T cells *in vitro* and *in vivo*.

## Results

### The response to QI9/A24 involves TCR diversity across vaccinated and convalescent donors

To determine the QI9/A24-specific TCR repertoire *ex vivo*, we performed single-cell sorting with the QI9/A24 tetramer in three HLA-A*24:02^+^-vaccinated and convalescent donors (**Table 1**, **Supplementary Figure 1A**) (9). This analysis revealed considerable clonotypic diversity in the response to the QI9/A24, with a total of 219 clonotypes isolated and 169 αβ TCR pairs (**Table 2**). Among them, the QI9/A24-specific CD8^+^ T cells predominantly expressed TCR clonotypes utilizing TRAV19*01 and TRBV20-1*01 in two vaccinated donors (GV33V and GV36V). However, the TCR clonotypes were highly diverse in convalescents, and dominant clonotypes, such as TRAV19*01/TRBV20-1*01, were rarely detected in other vaccinated donors and convalescents (**Table 2**). These data suggest that a diverse TCR repertoire characterizes the immunodominant T-cell response in HLA-A*24:02^+^ vaccinated and convalescent donors.

**Table 1:** Clinical samples used in this study, related to **Table 2** and **Figure 3**.

Cohort	Donor ID	Sex	Age	HLA-A24?	No. of doses	Dose				Days since last shot	COVID-19 severity	Sampling days post PCR positivity or onset	Blood collection
						1	2	3	4				
TCR repertoire													
A24+ vaccinated donors	GV33V	Male	40	Positive	x2	BNT162b2	BNT162b2	NA	NA	24	NA	NA	05/07/2021
	GV34V	Female	39	Positive	x2	BNT162b2	BNT162b2	NA	NA	24			05/07/2021
	GV36V	Male	40	Positive	x2	BNT162b2	BNT162b2	NA	NA	24			05/07/2021
A24+ COVID-19 convalescents	GV38C	Male	23	Positive	NA	NA	NA	NA	NA	NA	Mild	18	20/05/2021
	GV41C	Male	33	Positive	NA	NA	NA	NA	NA	NA	Mild	16	20/05/2021
	GV42C	Male	25	Positive	NA	NA	NA	NA	NA	NA	Mild	32	03/06/2021
*In vitro* stimulation													
A24+ vaccinated donors	GV15V	Female	24	Positive	x3	BNT162b2	BNT162b2	mRNA-1273	NA	39	NA	NA	07/03/2022
	GV16V	Male	23	Positive	x3	BNT162b2	BNT162b2	mRNA-1273	NA	43			11/03/2022
	GV24V	Male	24	Positive	x3	BNT162b2	BNT162b2	mRNA-1273	NA	28			08/03/2022
	GV33V	Male	40	Positive	x3	BNT162b2	BNT162b2	mRNA-1273	NA	71			08/04/2022
	GV36V	Male	40	Positive	x3	BNT162b2	BNT162b2	mRNA-1273	NA	21			02/03/2022
	GV59V	Male	38	Positive	x3	BNT162b2	BNT162b2	BNT162b2	NA	25			11/05/2022
	GV75V	Male	37	Positive	x3	mRNA-1273	mRNA-1273	mRNA-1273	NA	25			06/06/2022
A24+ COVID-19 convalescents	GV33 C-1	Male	40	Positive	x3	BNT162b2	BNT162b2	mRNA-1273	NA	477	Mild	9	11/10/2022
	GV33 C-2	Male	40	Positive	x3	BNT162b2	BNT162b2	mRNA-1273	NA	931	Mild	453	29/12/2023
	GV36C	Male	40	Positive	x3	BNT162b2	BNT162b2	mRNA-1273	NA	175	Mild	15	03/08/2022
	GV59C	Male	39	Positive	x4	BNT162b2	BNT162b2	BNT162b2	BNT162b2	290	Mild	12	01/08/2023
	GV75C	Male	38	Positive	x3	mRNA-1273	mRNA-1273	mRNA-1273	NA	177	Mild	38	05/12/2022
A24- vaccinated donors	GV25V	Male	24	Negative	x3	BNT162b2	BNT162b2	mRNA-1273	NA	NA	NA	NA	10/03/2022
	GV27V	Female	24	Negative	x3	BNT162b2	BNT162b2	mRNA-1273	NA				10/03/2022
	GV53V	Female	26	Negative	x3	BNT162b2	BNT162b2	BNT162b2	NA				08/04/2022
	GV74V	Female	34	Negative	x3	BNT162b2	BNT162b2	BNT162b2	NA				24/05/2022

NA, not applicable..

**Table 2:** TCR repertoire of QI9/A24-specific T cells .

Donor	TCRα			TCRβ				Count	Frequency	TCR
	TRAV	TRAJ	CDR3α	TRBV	TRBD	TRBJ	CDR3β			
GV33V	TRAV19*01	TRAJ33*01	CALSVLPGDSNYQLIW	TRBV20-1*01	TRBJ2-7*01	TRBD2*02	CSAEGTKNYEQYF	7	26.9	GV33 #7-8
	TRAV8-6*02	TRAJ21*01	CAVTGGGNFNKFYF	TRBV20-1*02	TRBJ2-7*01	TRBD1*01	CSARDLGQAYEQYF	3	11.5	GV33 #1
	TRAV3*01	TRAJ8*01	CAGVLFNTGFQKLVF	TRBV20-1*02	TRBJ2-1*01	TRBD2*01	CSASDRGASGSFSNEQFF	3	11.5	GV33 #57
	TRAV16*01	TRAJ6*01	CALSAPRGGGSYIPTF	TRBV5-6*01	TRBJ2-7*01	TRBD1*01	CASRGSGGYSEQYF	2	7.7	
	TRAV12-2*01	TRAJ4*01	CAAEGLSGGYNKLIF	TRBV7-9*03	TRBJ2-7*01	TRBD2*01	CASGTSGYEQYF	2	7.7	
	TRAV13-2*01	TRAJ39*01	CAEEAGNMLTF	TRBV6-6*01	TRBJ2-1*01	TRBD1*01	CASSYRAYYNEQFF	2	7.7	
	TRAV24*01	TRAJ58*01	CAKGETSGSRLTF	TRBV5-6*01	TRBJ2-1*01	TRBD2*01	CASSLLAALTLGQFF	2	7.7	
	TRAV8-2*03	TRAJ42*01	CVVSDLYGGSQGNLIF	TRBV20-1*01	TRBJ2-7*01	TRBD2*01	CSARDVSGGRHYEQYF	2	7.7	
	TRAV3*01	TRAJ8*01	CAGLEYNPGPKAFAP	TRBV20-1*02	TRBJ2-1*01		CSASDRGASGSFSNEQFF	1	3.8	
	TRAV24*01	TRAJ43*01	CASSWADMRF	TRBV5-6*01	TRBJ1-1*01	TRBD1*01	CASSLAGLNTEAFF	1	3.8	
	TRAV17*01	TRAJ30*01	CATDAWRNRDDKIIF	TRBV7-2*02	TRBJ2-5*01		CASSLERFGRHQETQYF	1	3.8	
GV34V	TRAV21*01	TRAJ43*01	CAAPRYNNNDMRF	TRBV2*01	TRBJ2-2*01	TRBD1*01	CASSEGADAGELFF	6	11.1	GV34 #43
	TRAV13-2*01	TRAJ52*01	CPGGTSYGKLTF	TRBV20-1*02	TRBJ2-7*01	TRBD2*01	CSARGTSSSYEQYF	5	9.3	GV34 #34-6
	TRAV8-6*02	TRAJ16*02	CAVSDLGQKLLF	TRBV5-6*01	TRBJ2-1*01	TRBD2*01	CASSMSGGSEQFF	4	7.4	
	TRAV34*01	TRAJ45*01	CGAANSGGGADGLTF	TRBV2*01	TRBJ1-1*01	TRBD1*01	CASSLQFAGTEAFF	4	7.4	
	TRAV21*01	TRAJ33*01	CTARDSTYQLIW	TRBV15*02	TRBJ2-7*01	TRBD1*01	CATSTPGEQYF	3	5.6	
	TRAV8-6*02	TRAJ21*01	CAVTPVYNFNKFYF	TRBV20-1*01	TRBJ2-7*01	TRBD1*01	CSARDLGQAYEQYF	2	3.7	
	TRAV2*01	TRAJ30*01	CAVEDRDDKIIF	TRBV20-1*02	TRBJ2-7*01	TRBD2*02	CSARDPRLAGVEQYF	2	3.7	
	TRAV14/DV4*03	TRAJ47*01	CAMREQIGGNKLVF	TRBV7-9*01	TRBJ2-5*01	TRBD1*01	CASSASGTAQETQYF	1	1.9	
	TRAV22*01	TRAJ44*01	CAVVINTGTASKLTF	TRBV2*01	TRBJ2-7*01	TRBD2*02	CASSEGRWVYEQYF	1	1.9	
	TRAV8-6*01	TRAJ17*01	CAVIHIKAAGNKLTF	TRBV19*01	TRBJ2-7*01	TRBD1*01	CASSISMLREQYF	1	1.9	
	TRAV3*01	TRAJ10*01	CAVKVLTGGGNKLTF	TRBV27*01	TRBJ2-7*01	TRBD2*01	CASSLGGGLYEQYF	1	1.9	
	TRAV8-6*02	TRAJ57*01	CAVSGEGGSEKLVF	TRBV5-4*01	TRBJ1-2*01	TRBD1*01	CASSLKGTLYGYTF	1	1.9	
	TRAV16*01	TRAJ32*01	CALSGATNKLIF	TRBV5-6*01	TRBJ2-1*01		CASSLNNHNEQFF	1	1.9	
	TRAV8-4*01	TRAJ50*01	CAVKDYDKVIF	TRBV5-6*01	TRBJ1-5*01	TRBD1*01	CASSLNRGNQPQHF	1	1.9	
	TRAV13-2*01	TRAJ15*01	CAENPAGTALIF	TRBV7-3*01	TRBJ1-2*01	TRBD2*01	CASSLSAGDGYTF	1	1.9	
	TRAV24*01	TRAJ52*01	CAFCGGTSYGKLTF	TRBV5-6*01	TRBJ2-5*01	TRBD1*01	CASSLSGTGGETQYF	1	1.9	
	TRAV8-6*01	TRAJ54*01	CAVRQGAQKLVF	TRBV5-6*01	TRBJ2-7*01	TRBD1*01	CASSLVSQVYEQYF	1	1.9	
	TRAV8-3*01	TRAJ39*01	CAVSHAGNMLTF	TRBV27*01	TRBJ2-5*01	TRBD2*01	CASSPGLAGIQETQYF	1	1.9	
	TRAV14/DV4*02	TRAJ27*01	CAIYINTNAGKSTF	TRBV27*01	TRBJ2-3*01	TRBD1*01	CASSPTPTGADTQYF	1	1.9	
	TRAV29/DV5*04	TRAJ39*01	CAAIPNNAGNMLTF	TRBV4-1*01	TRBJ1-2*01	TRBD1*01	CASSQVYGPTNYGYTF	1	1.9	
	TRAV29/DV5*04	TRAJ54*01	CAAPLSQKLVF	TRBV5-6*01	TRBJ1-5*01	TRBD1*01	CASSRTGGGQPQHF	1	1.9	
	TRAV27*01	TRAJ54*01	CAGARGAQKLVF	TRBV5-1*01	TRBJ1-1*01	TRBD1*01	CASSSWGAGTEAFF	1	1.9	
	TRAV12-2*01	TRAJ49*01	CAAYNTNTGNQFYF	TRBV11-2*01	TRBJ2-1*01	TRBD2*01	CASSTTSWDEQFF	1	1.9	
	TRAV4*01	TRAJ6*01	CLVGDVSGGSYIPTF	TRBV9*01	TRBJ1-2*01	TRBD2*02	CASSVAETEYGYTF	1	1.9	
	TRAV4*01	TRAJ45*01	CLVGDRTGGGADGLTF	TRBV5-1*01	TRBJ1-2*01	TRBD1*01	CASSWGTGSGGYTF	1	1.9	
	TRAV21*01	TRAJ26*01	CAARDNYGQNFVF	TRBV6-2*01	TRBJ2-7*01	TRBD1*01	CASSYSRSRGDYEQYF	1	1.9	
	TRAV27*01	TRAJ41*01	CAGAIPNSGYALNF	TRBV6-5*01	TRBJ1-1*01	TRBD1*01	CASSYYDGDTEAFF	1	1.9	
	TRAV12-2*02	TRAJ44*01	CAVTGTASKLTF	TRBV2*01	TRBJ2-7*01	TRBD1*01	CASTEQGVGQYF	1	1.9	
	TRAV8-6*02	TRAJ52*01	CAASTTSYGKLTF	TRBV15*02	TRBJ1-2*01	TRBD1*01	CATEGIEGGYDYGYTF	1	1.9	
	TRAV12-1*01	TRAJ22*01	CVGSGSARQLTF	TRBV15*02	TRBJ2-7*01	TRBD2*02	CATSTGSSSYEQYF	1	1.9	
	TRAV6*07	TRAJ12*01	CPRQSWMDSSYKLIF	TRBV30*01	TRBJ2-7*01	TRBD1*01	CAWSIQSGSYEQYF	1	1.9	
	TRAV19*01	TRAJ39*01	CALWCWRGNAGNMLTF	TRBV20-1*01	TRBJ2-5*01	TRBD1*01	CSANREVSQETQYF	1	1.9	
	TRAV23/DV6*01	TRAJ10*01	CAAYLATGGGNKLTF	TRBV20-1*01	TRBJ2-3*01	TRBD1*01	CSARDLGGTGADTQYF	1	1.9	
	TRAV23/DV6*01	TRAJ5*01	CAASIKLMDTGRRALTF	TRBV20-1*02	TRBJ1-4*01	TRBD1*01	CSARGFGNEKLFF	1	1.9	
	TRAV12-1*01	TRAJ4*01	CVVISDSGGYNKLIF	TRBV20-1*02	TRBJ2-7*01	TRBD2*01	CSARGTSGAYEQYF	1	1.9	
GV36V	TRAV19*01	TRAJ9*01	CALSEPPSGGFKTIF	TRBV20-1*01	TRBJ1-1*01	TRBD1*01	CSARESGENTEAFF	8	30.8	GV36 #11
	TRAV19*01	TRAJ9*01	CALSEPPSGGFKTIF	TRBV20-1*01	TRBJ1-1*01	TRBD1*01	CSARGQGLNTEAFF	2	7.7	GV36 #10-2
	TRAV8-4*03	TRAJ47*02	CAVSEVGNKLVF	TRBV5-6*01	TRBJ2-1*01	TRBD2*01	CASSSSGGGEQFF	4	15.4	
	TRAV23/DV6*01	TRAJ22*01	CAAPFITSARQLTF	TRBV5-6*01	TRBJ2-7*01	TRBD2*01	CASSLAGAGEQYF	3	11.5	
	TRAV8-6*01	TRAJ20*01	CAVSPLGDKLSF	TRBV5-6*01	TRBJ2-2*01	TRBD2*01	CASRINSAGELFF	1	3.8	
	TRAV2*01	TRAJ6*01	CAVEDSSGGSYIPTF	TRBV20-1*01	TRBJ2-7*01	TRBD2*01	CSARDEESAYEQYF	1	3.8	
	TRAV8-6*01	TRAJ21*01	CAVTPIYNFNKFYF	TRBV20-1*01	TRBJ2-7*01	TRBD1*01	CSARDLGQAYEQYF	1	3.8	
	TRAV14/DV4*02	TRAJ57*01	CAMREGHERGGSEKLVF	TRBV9*01	TRBJ2-1*01	TRBD1*01	CASGRRNEQFF	1	3.8	
	TRAV10*01	TRAJ37*01	CVVSPTGKLIF	TRBV6-5*01	TRBJ2-5*01	TRBD1*01	CASSFLQGSEETQYF	1	3.8	
	TRAV4*01	TRAJ20*01	CLVGPPSNDYKLSF	TRBV27*01	TRBJ2-7*01	TRBD2*02	CASSPAPPGVYEQYF	1	3.8	
	TRAV39*01	TRAJ58*01	CAVDIETSGSRLTF	TRBV9*01	TRBJ2-6*01	TRBD2*01	CASSPGGYGPGANVLTF	1	3.8	
	TRAV1-1*02	TRAJ10*01	CAVRAITGGGNKLTF	TRBV5-1*01	TRBJ2-5*01	TRBD2*01	CASSQSSEGNQETQYF	1	3.8	
	TRAV41*01	TRAJ34*01	CAVRSYNTDKLIF	TRBV9*01	TRBJ2-2*01	TRBD2*01	CASTGGDSYYGELFF	1	3.8	
	TRAV8-6*012	TRAJ48*01	CAVSVLGEKLTF	TRBV5-6*01	TRBJ2-1*01	TRBD2*01	CASSLAGGGEQFF	clone	n.a	GV36 8C6
GV38C	TRAV24*01	TRAJ52*01	CAFSGGTSYGKLTF	TRBV5-6*01	TRBJ2-7*01	TRBD1*01	CASSTGTLVYEQYF	2	5.4	
	TRAV12-1*01	TRAJ16*02	CAVSPFSDGQKLLF	TRBV20-1*01	TRBJ2-1*01	TRBD2*01	CSARDYGLAGVEQFF	1	2.6	
	TRAV14/DV4*02	TRAJ5*01	CAMRLQHTGRRALTF	TRBV20-1*01	TRBJ2-1*01	TRBD2*01	CSARRPGIPYNEQFF	1	2.6	
	TRAV12-2*02	TRAJ4*01	CAVRPGGYNKLIF	TRBV12-3*01	TRBJ2-2*01	TRBD1*01	CASGARLVFPGELFF	1	2.6	
	TRAV29/DV5*01	TRAJ45*01	CAASASGGGADGLTF	TRBV5-8*01	TRBJ1-1*01	TRBD1*01	CASPPTDNTEAFF	1	2.6	
	TRAV16*01	TRAJ30*01	CAVMNRDDKIIF	TRBV9*01	TRBJ2-3*01	TRBD1*01	CASSEISGDTQYF	1	2.6	
	TRAV8-6*01	TRAJ3*01	CAVTELSSASKIIF	TRBV25-1*01	TRBJ2-7*01	TRBD1*01	CASSELGSPSSYEQYF	1	2.6	
	TRAV24*01	TRAJ53*01	CAFSGGSNYKLTF	TRBV5-6*01	TRBJ1-6*02	TRBD1*01	CASSFGGQVYNSPLHF	1	2.6	
	TRAV3*01	TRAJ29*01	CAVRDSGNTPLVF	TRBV12-4*01	TRBJ1-1*01	TRBD2*01	CASSFGGWNTEAFF	1	2.6	
	TRAV21*01	TRAJ4*01	SAKRAGGYNKLIF	TRBV27*01	TRBJ2-5*01	TRBD2*01	CASSFRYQETQYF	1	2.6	
	TRAV8-6*01	TRAJ48*01	CAVSPGEKLTF	TRBV28*01	TRBJ1-5*01	TRBD1*01	CASSFWDSNQPQHF	1	2.6	
	TRAV29/DV5*01	TRAJ34*01	CAASGGADKLIF	TRBV9*01	TRBJ1-6*01	TRBD1*01	CASSGNPGDRGPREVLHF	1	2.6	
	TRAV12-3*01	TRAJ20*01	VQRQQYPA#SNDYKLSF	TRBV28*01	TRBJ1-5*01	TRBD1*01	CASSILGEVRGWVDQPQHF	1	2.6	
	TRAV41*01	TRAJ34*01	CAVRAYNTDKLIF	TRBV5-1*01	TRBJ1-4*01	TRBD1*01	CASSLAGQAVATNEKLFF	1	2.6	
	TRAV5*01	TRAJ9*01	CAESTGGFKTIF	TRBV28*01	TRBJ2-2*01	TRBD2*02	CASSLELAGVYTGELFF	1	2.6	
	TRAV19*01	TRAJ49*01	CALSEVNPGTQFYF	TRBV7-6*01	TRBJ2-1*01	TRBD2*01	CASSLGGAYNEQFF	1	2.6	
	TRAV3*01	TRAJ53*01	CAVENSGGSNYKLTF	TRBV7-3*01	TRBJ2-7*01	TRBD2*01	CASSLGGVPSLYEQYF	1	2.6	
	TRAV4*01	TRAJ20*01	CLVSSGYKLSF	TRBV7-3*01	TRBJ2-1*01	TRBD2*02	CASSLIAGVTYEQFF	1	2.6	
	TRAV29/DV5*01	TRAJ57*01	CAASTTQGGSEKLVF	TRBV11-2*03	TRBJ2-7*01	TRBD2*01	CASSLLGLWPYEQYF	1	2.6	
	TRAV17*01	TRAJ58*01	CATEGEETSGSRLTF	TRBV27*01	TRBJ2-7*01	TRBD1*01	CASSLQWGYYEQYF	1	2.6	
	TRAV13-1*02	TRAJ45*01	CASIRPPGGGADGLTF	TRBV13*01	TRBJ2-4*01	TRBD1*01	CASSLREWGKNIQYF	1	2.6	
	TRAV14/DV4*01	TRAJ30*01	CAMREDRDDKIIF	TRBV28*01	TRBJ2-7*01	TRBD2*01	CASSLSPGLTPEQYF	1	2.6	
	TRAV29/DV5*01	TRAJ40*01	CAASARSSGANQFNF	TRBV12-4*01	TRBJ2-2*01	TRBD1*01	CASSLTRRLGPNTGELFF	1	2.6	
	TRAV21*01	TRAJ58*01	CAVPPLRETSGSRLTF	TRBV6-5*01	TRBJ2-7*01	TRBD1*01	CASSQTGFIYEQYF	1	2.6	
	TRAV8-6*01	TRAJ23*01	CAVSDKNNQGGKLIF	TRBV3-1*01	TRBJ2-7*01	TRBD2*02	CASSQVLLAGGSSYEQYF	1	2.6	
	TRAV22*01	TRAJ52*01	CAVEYAGGTSYGKLTF	TRBV6-5*01	TRBJ2-1*01	TRBD1*01	CASSSMWGQKDNEQFF	1	2.6	
	TRAV12-1*01	TRAJ8*01	CAVNRRDTGNTGFQKLVF	TRBV12-4*01	TRBJ2-7*01	TRBD1*01	CASSSPGAEQYF	1	2.6	
	TRAV3*01	TRAJ35*01	CAVRDKASFGNVLHC	TRBV6-5*01	TRBJ2-1*01	TRBD1*01	CASSSPSRRDWGPFYYNEQFF	1	2.6	
	TRAV41*01	TRAJ34*01	CAVLSYNTDKLIF	TRBV11-3*04	TRBJ2-5*01	TRBD1*01	CASSSQEPGGSRETQYF	1	2.6	
	TRAV16*01	TRAJ34*01	CAPPFYNTDKLIF	TRBV6-2*01	TRBJ2-1*01	TRBD1*01	CASSWTDREQFF	1	2.6	
	TRAV14/DV4*02	TRAJ33*01	CAMREFDSNYQLIW	TRBV6-2*01	TRBJ1-1*01	TRBD2*02	CASSYFFRGEATEAFF	1	2.6	
	TRAV21*01	TRAJ34*01	CAVRSFYNTDKLIF	TRBV6-5*01	TRBJ2-5*01	TRBD1*01	CASSYVGQGQETQYF	1	2.6	
	TRAV6*02	TRAJ36*01	CALKTGANNLFF	TRBV28*01	TRBJ1-2*01	TRBD2*02	CASTPGREWGPGLYTF	1	2.6	
	TRAV17*01	TRAJ52*01	CATDLTPRNAGGTSYGKLTF	TRBV24-1*01	TRBJ2-1*01	TRBD1*01	CATSDWQTGHRLNEQFF	1	2.6	
	TRAV12-2*02	TRAJ42*01	CAVNSPFNYGGSQGNLIF	TRBV24-1*01	TRBJ2-1*01	TRBD1*01	CATSERTGGSYNEQFF	1	2.6	
	TRAV38-1*01	TRAJ47*01	CAFMKYDGNKLVF	TRBV29-1*01	TRBJ2-7*01	TRBD2*02	CSVDLSPPSGRQVGRYEQYF	1	2.6	
GV41C	TRAV38-2/DV8*01	TRAJ34*01	CAYNTDKLIF	TRBV6-2*01	TRBJ2-7*01	TRBD2*02	CASSRLAGGSAYEQYF	2	11.1	
	TRAV19*01	TRAJ9*01	CALSEPPTGGFKTIF	TRBV20-1*01	TRBJ1-1*01	TRBD2*01	CSARNLGGNTEAFF	2	11.1	
	TRAV19*01	TRAJ9*01	CALSEPPSGGFKTIF	TRBV20-1*01	TRBJ1-1*01	TRBD1*01	CSARGQGLNTEAFF	1	5.6	
	TRAV22*01	TRAJ40*01	CAVTHLSSGTYKYIF	TRBV20-1*01	TRBJ2-7*01	TRBD1*01	CSARDPGQAYEQYF	1	5.6	
	TRAV12-3*01	TRAJ52*01	CAMIEAGGTSYGKLTF	TRBV20-1*01	TRBJ1-5*01	TRBD2*01	CSARDLYGQPQHF	1	5.6	
	TRAV29/DV5*04	TRAJ26*01	CAASAYGQNFVF	TRBV20-1*01	TRBJ1-2*01	TRBD1*01	CSAREMDVAGPLVHGYTF	1	5.6	
	TRAV24*01	TRAJ53*01	CAPPKGGSNYKLTF	TRBV20-1*01	TRBJ2-7*01	TRBD2*02	CSARRIEGGGHNEQYF	1	5.6	
	TRAV4*01	TRAJ3+D107:D1082*02	CLVDGGATNKLIF	TRBV5-1*01	TRBJ1-1*01	TRBD1*01	CASRQVGTEAFF	1	5.6	
	TRAV21*01	TRAJ21*01	CAVNLYNFNKFYF	TRBV6-1*01	TRBJ2-5*01	TRBD1*01	CASSDGEGGIFQETQYF	1	5.6	
	TRAV4*01	TRAJ13*02	CLVGLSGGYQKVTF	TRBV2*01	TRBJ2-1*01	TRBD2*01	CASSDGTSGGKNEQFF	1	5.6	
	TRAV8-1*01	TRAJ37*01	CAVGSGNTGKLIF	TRBV10-2*01	TRBJ2-7*01		CASSDTPSPHEQYF	1	5.6	
	TRAV41*01	TRAJ34*01	CAVASYNTDKLIF	TRBV5-6*01	TRBJ1-3*01	TRBD1*01	CASSFRHGASGNTIYF	1	5.6	
	TRAV17*01	TRAJ5*01	CATDAWTGRRALTF	TRBV27*01	TRBJ2-5*01	TRBD1*01	CASSLQGSETQYF	1	5.6	
	TRAV13-1*01	TRAJ36*01	CAVRRETGANNLFF	TRBV5-1*01	TRBJ1-2*01	TRBD2*02	CASSLVWEAGYTF	1	5.6	
	TRAV8-6*01	TRAJ40*01	CAVTPGTYKYIF	TRBV11-2*01	TRBJ1-1*01	TRBD1*01	CASSTQGGRPEAFF	1	5.6	
	TRAV4*01	TRAJ6*01	CLVGPLSGGSYIPTF	TRBV6-5*01	TRBJ1-1*01	TRBD1*01	CASSYSEGNPGEAFF	1	5.6	
GV42C	TRAV8-2*01	TRAJ3*01	CVVIMYSSASKIIF	TRBV10-3*03	TRBJ2-7*01	TRBD1*01	CAISDPGQGPYEQYF	1	1.8	
	TRAV10*01	TRAJ4*01	CVVHEPEAYNNLIF	TRBV10-3*03	TRBJ2-4*01	TRBD2*01	CAISESTLPARGGKNIQYF	1	1.8	
	TRAV4*01	TRAJ15*01	CLVVSWVQAGTALIF	TRBV28*01	TRBJ1-1*01	TRBD1*01	CASQLGGRDTEAFF	1	1.8	
	TRAV38-1*03	TRAJ45*01	CAFMKLAGGGADGLPF	TRBV2*01	TRBJ2-7*01	TRBD2*01	CASRRLVRPTYEQYF	1	1.8	
	TRAV14/DV4*01	TRAJ43*01	CAMREVYNDMRF	TRBV4-1*01	TRBJ1-1*01	TRBD1*01	CASRTRDRVGTEAFF	1	1.8	
	TRAV24*01	TRAJ45*01	CAFIDSGGGADGLTF	TRBV2*01	TRBJ2-1*01	TRBD2*02	CASSDKAGGRDEQFF	1	1.8	
	TRAV41*01	TRAJ34*01	CAARSYNTDKLIF	TRBV6-2*01	TRBJ2-2*01	TRBD2*02	CASSDTQLAGNTGELFF	1	1.8	
	TRAV8-3*01	TRAJ34*01	CAVGEFWNTDKLIF	TRBV2*01	TRBJ2-6*01	TRBD1*01	CASSEFGTGVAGANVLTF	1	1.8	
	TRAV12-1*01	TRAJ8*01	CVVNKRSTGFQKLVF	TRBV6-1*01	TRBJ2-7*01	TRBD1*01	CASSENWGYEQYF	1	1.8	
	TRAV21*01	TRAJ54*01	CAVRRLQGAQKLVF	TRBV7-8*01	TRBJ2-2*01	TRBD1*01	CASSFQGYTGELFF	1	1.8	
	TRAV8-1*01	TRAJ30*01	CAVNGMNRDDKITF	TRBV11-3*04	TRBJ2-7*01	TRBD1*01	CASSFRGISYEQYF	1	1.8	
	TRAV8-4*01	TRAJ48*01	CAVSPSNFGNEKLTF	TRBV28*01	TRBJ2-1*01	TRBD2*02	CASSFWVAGGITYNEQFF	1	1.8	
	TRAV5*01	TRAJ44*01	CAEYYTGTASKLTF	TRBV7-6*01	TRBJ2-3*01	TRBD1*01	CASSFYEGHTQYF	1	1.8	
	TRAV12-2*02	TRAJ3*01	CAGWASSASKIIF	TRBV9*01	TRBJ2-7*01	TRBD2*01	CASSGGLAGTIYEQYF	1	1.8	
	TRAV13-1*02	TRAJ43*01	CAASIRRGDMRF	TRBV9*01	TRBJ2-2*01	TRBD2*02	CASSGGRTKNTGELFF	1	1.8	
	TRAV1-2*03	TRAJ35*01	CAVSWGFGNVLHC	TRBV3-1*01	TRBJ2-1*01	TRBD1*01	CASSHVSYNEQFF	1	1.8	
	TRAV38-2/DV8*01	TRAJ52*01	CAYRSAFAGGTSYGKLTF	TRBV11-2*01	TRBJ2-1*01	TRBD1*01	CASSLAAGVPGFFYNEQFF	1	1.8	
	TRAV8-3*01	TRAJ41*01	CAVDTGSGYALNF	TRBV5-6*01	TRBJ1-2*01	TRBD1*01	CASSLAWDGYTF	1	1.8	
	TRAV8-2*01	TRAJ26*01	CVVNYGQNFVF	TRBV12-4*01	TRBJ1-6*01	TRBD2*02	CASSLCVEDGCSYNSPLHF	1	1.8	
	TRAV9-2*01	TRAJ23*01	CALNRGVQGGKLIF	TRBV5-1*01	TRBJ2-7*01	TRBD1*01	CASSLDFTGPSYEQYF	1	1.8	
	TRAV12-2*02	TRAJ13*02	CASPSGGYQKVTF	TRBV5-1*01	TRBJ1-2*01	TRBD1*01	CASSLDLLRDSYYGYTF	1	1.8	
	TRAV3*01	TRAJ32*02	CAVRDMEGVYGGATNKLIF	TRBV5-1*01	TRBJ2-5*01	TRBD2*02	CASSLDSRDRTRYQETQYF	1	1.8	
	TRAV17*01	TRAJ53*01	CATEPNSGGSNYKLTF	TRBV7-6*01	TRBJ2-7*01	TRBD2*01	CASSLEPPLVGRLEQYF	1	1.8	
	TRAV19*01	TRAJ23*01	CALSEAEHILWGKLIF	TRBV28*01	TRBJ2-7*01	TRBD1*01	CASSLFPRGLASYEQYF	1	1.8	
	TRAV4*01	TRAJ45*01	CLVGPPRGYSGGGADGLTF	TRBV5-6*01	TRBJ2-1*01	TRBD1*01	CASSLGIEQFF	1	1.8	
	TRAV19*01	TRAJ45*01	CALRCAGGGADGLTF	TRBV7-9*03	TRBJ1-1*01	TRBD1*01	CASSLLAGYNTEAFF	1	1.8	
	TRAV27*01	TRAJ57*01	CAGARGGGSEKLVF	TRBV9*01	TRBJ2-7*01	TRBD2*01	CASSLSGTSFHEQYF	1	1.8	
	TRAV12-2*02	TRAJ13*02	CAIEKRGYQKVTF	TRBV6-5*01	TRBJ2-7*01	TRBD2*01	CASSPAQLSGVYEQYF	1	1.8	
	TRAV19*01	TRAJ53*01	CALGGSNYKLTF	TRBV27*01	TRBJ2-6*01	TRBD1*01	CASSPLTRTGANVLTF	1	1.8	
	TRAV12-2*02	TRAJ23*01	CAVPIYNQGGKLIF	TRBV18*01	TRBJ1-3*01	TRBD1*01	CASSPSGRSAKLNTIYF	1	1.8	
	TRAV19*01	TRAJ34*01	CALSEYYTDKLIF	TRBV18*01	TRBJ1-5*01	TRBD1*01	CASSPTGTGAFSWNQPQHF	1	1.8	
	TRAV3*01	TRAJ20*01	CAVRDIPNDYKLSF	TRBV12-4*01	TRBJ2-3*01	TRBD2*01	CASSPYGTDTQYF	1	1.8	
	TRAV29/DV5*04	TRAJ20*01	CAASANDYKLSF	TRBV4-1*01	TRBJ2-7*01	TRBD2*02	CASSQDELGGAYEQYF	1	1.8	
	TRAV8-6*02	TRAJ39*01	CPVKHNTGNMLTF	TRBV4-1*01	TRBJ2-7*01	TRBD1*01	CASSQDLTGRGSYEQYF	1	1.8	
	TRAV12-3*01	TRAJ35*01	CAMEGSFGNVLHC	TRBV3-1*01	TRBJ2-7*01	TRBD2*01	CASSQDNMRLSYEQYF	1	1.8	
	TRAV10*01	TRAJ42*01	CVVSDVNYGGSQGNLIF	TRBV5-1*01	TRBJ2-7*01	TRBD1*01	CASSSNRGRGGSYEQYF	1	1.8	
	TRAV25*01	TRAJ53*01	CAGPGSGGSNYKLTF	TRBV9*01	TRBJ2-7*01	TRBD2*01	CASSVAPGSYEQYF	1	1.8	
	TRAV41*01	TRAJ42*01	CAVRRAYGGSQGNLIF	TRBV9*01	TRBJ2-6*01	TRBD1*01	CASSVASGQGRSGANVLTF	1	1.8	
	TRAV2*01	TRAJ40*01	CASTSGTYKYIF	TRBV9*01	TRBJ2-2*01	TRBD2*01	CASSVFGTSESGELFF	1	1.8	
	TRAV39*01	TRAJ45*01	CAVDIESGGGADGLTF	TRBV9*01	TRBJ2-7*01	TRBD1*01	CASSVTERTGGEEQYF	1	1.8	
	TRAV19*01	TRAJ57*01	CAVEGQGGSEKLVF	TRBV9*01	TRBJ2-2*01	TRBD2*01	CASSVVVTPNTGELFF	1	1.8	
	TRAV29/DV5*01	TRAJ49*01	CAASTGNQFYF	TRBV5-4*01	TRBJ2-3*01	TRBD2*02	CASSYKLAGVEDTQYF	1	1.8	
	TRAV38-2/DV8*01	TRAJ57*01	CAYRSAEVSEKLVF	TRBV6-5*01	TRBJ2-3*01	TRBD1*01	CASSYRYGARDVIADTQYF	1	1.8	
	TRAV29/DV5*04	TRAJ30*01	CAASFMNRDDKIIF	TRBV6-6*01	TRBJ1-2*01	TRBD2*01	CASSYSAGSYGYTF	1	1.8	
	TRAV4*01	TRAJ29*01	CLLASGNTPLVF	TRBV5-1*01	TRBJ2-3*01	TRBD1*01	CASSYTGTGVQDTQYF	1	1.8	
	TRAV29/DV5*01	TRAJ52*01	CAASAVGAGGTSYGKLTF	TRBV24-1*01	TRBJ1-1*01	TRBD2*01	CATSDSKGGSRNTEAFF	1	1.8	
	TRAV8-2*01	TRAJ48*01	CVVSPFLFGNEKLTF	TRBV15*02	TRBJ2-1*01	TRBD1*01	CATSRDRTGAEQFF	1	1.8	
	TRAV10*01	TRAJ24*02	CVVRSSWGKLQF	TRBV24-1*01	TRBJ2-1*01	TRBD2*01	CATSVSLVGEQFF	1	1.8	
	TRAV26-2*01	TRAJ39*01	CIPDGNMLTF	TRBV30*01	TRBJ2-7*01	TRBD1*01	CAWSVDRGQQSYEQYF	1	1.8	
	TRAV29/DV5*04	TRAJ34*01	CAASSTDKLIF	TRBV20-1*01	TRBJ2-1*01	TRBD2*01	CSAFSGDRSYNEQFF	1	1.8	
	TRAV38-2/DV8*01	TRAJ40*01	CAFIAGTYKYIF	TRBV20-1*01	TRBJ1-1*01	TRBD1*01	CSAPEDRNTEAFF	1	1.8	
	TRAV12-2*02	TRAJ43*01	CAVNIWGNDMRF	TRBV20-1*01	TRBJ2-1*01	TRBD1*01	CSARDRADNNEQFF	1	1.8	
	TRAV41*01	TRAJ40*01	CAVSRITSGTYKYIF	TRBV20-1*01	TRBJ2-7*01	TRBD1*01	CSARDTGLSSGNPYEQYF	1	1.8	
	TRAV8-1*01	TRAJ31*01	CAVNEGARLMF	TRBV20-1*01	TRBJ2-7*01	TRBD1*01	CSAREWDPFEQYF	1	1.8	
	TRAV20*02	TRAJ42*01	CAVRGSQGNLIF	TRBV20-1*01	TRBJ1-1*01	TRBD1*01	CSARIKVSPLNTEAFF	1	1.8	
	TRAV21*02	TRAJ53*01	CAVRPRGGSGGSNYKLTF	TRBV29-1*01	TRBJ2-2*01	TRBD1*01	CSVDPSGQGGSGELFF	1	1.8	
	TRAV8-4*03	TRAJ42*01	CAVRDYGGSQGNLIF	TRBV29-1*01	TRBJ2-1*01	TRBD1*01	CSVETMGGFGFDEQFF	1	1.8	

### Cross-reactivity of QI9/A24-specific TCRs to variant peptides derived from other coronaviruses

**Figure 1:**
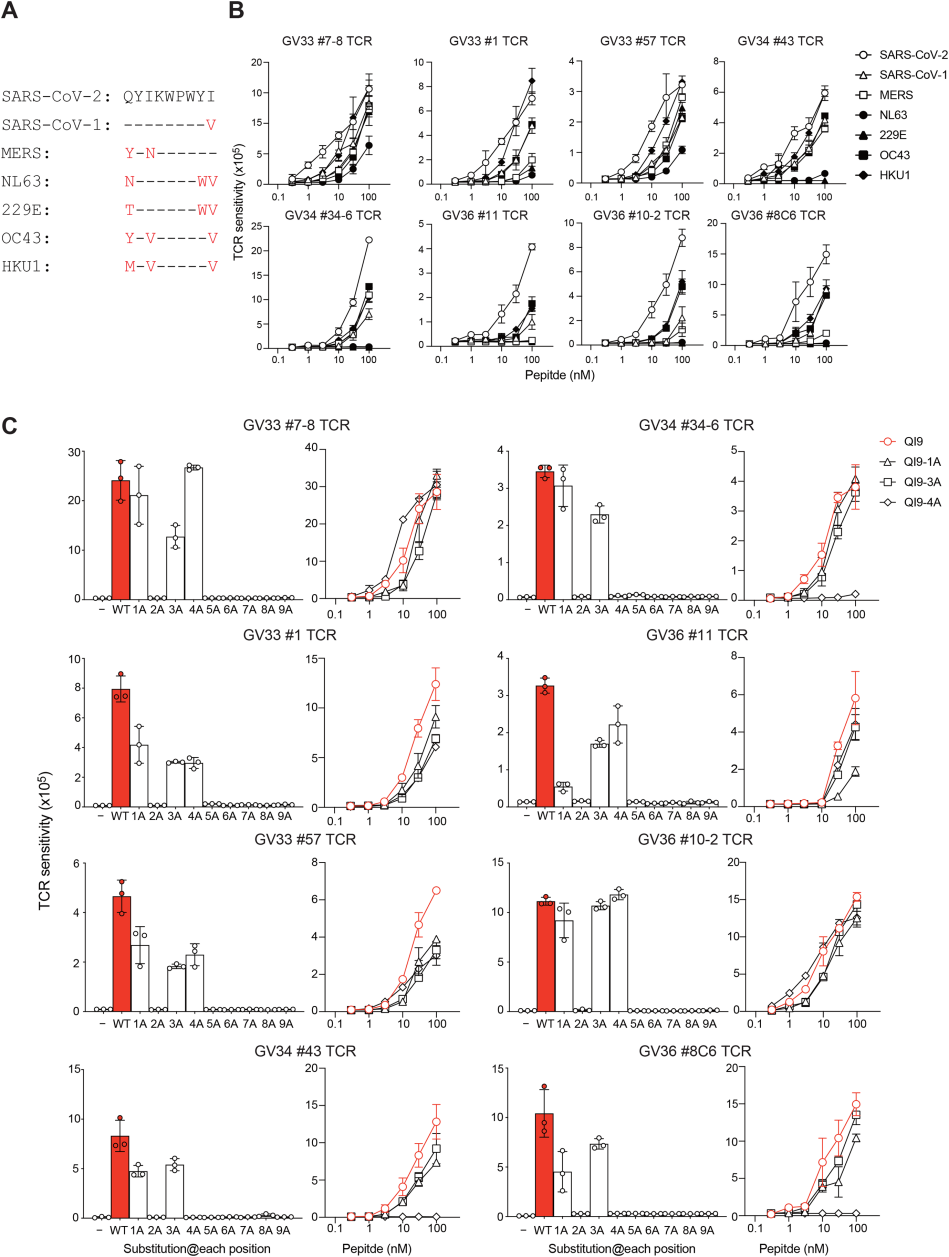
Cross-recognition of QI9/A24-specific TCRs toward variant peptides. **(A)**, Sequence similarity of the QI9/A24 peptide in SARS-CoV-1, MERS, and common cold coronaviruses. **(B)**, Peptide titration of TCR-transduced Jurkat cell lines. TCR cross-reactivity of TCR-reporter cells expressing QI9/A24-specific TCRs toward the variant peptides derived from SARS-CoV-1, MERS, and common cold coronaviruses. The TCR cross-reactivity was evaluated by NFAT-luciferase reporter activity in TCR-transduced Jurkat cells. **(C)**, Sensitivity of QI9/A24-specific TCRs toward a series of alanine substitutions in the QI9/A24 peptide backbone. TCR recognitions at 30 nM peptide (left panel) and peptide titration of the QI9, QI9-1A, QI9-3A, and QI9-4A variant peptides. TCR recognition of the QI9 peptide is shown in red. **(B, C)**, The assay was performed in triplicate, and the means are shown with the SD. Data are representative of three independent experiments.

Sequence alignment of the consensus SARS-CoV-2 QI9/A24 peptide with the corresponding SARS-CoV-1-derived QI9/A24 revealed a high degree of sequence similarity, differing by a single amino acid residue at position 9, and the MERS-derived QI9 peptide exhibited the similarity to the consensus sequence, sharing identity at only two positions, namely, the N-terminus and position 3 (**Figure 1A**) (11, 12, 15). Moreover, seasonal coronavirus-derived sequences shared homology with the QI9/A24 with differences at just two or three amino acid residues at either the N-terminus, or positions 3, 8, or 9. We first examined HLA binding of these variant peptides and found that all retained binding to HLA-A*24:02 with similar affinity (**Table 3**). To investigate TCR cross-reactivity toward variant peptides, we focused on eight TCR clonotypes dominant in each vaccinated donor (GV33V, GV34V, and GV36V) (**Table 2**). Pairs of TCR α and β chains from vaccinated donors were reconstituted in a TCR-deficient NFAT-luciferase reporter cell line (9). These QI9/A24-specific TCRs were expressed on the cell surface and bound cognate tetramers (**Supplementary Figure 1B**). The TCR cross-reactivity profiles varied among QI9/A24-specific TCR clonotypes (**Figure 1B**). However, all TCRs tested showed weaker recognition of variant peptides than the consensus peptide at low concentrations (<10 nM) (**Figure 1B**), suggesting that mRNA vaccination may revive preexisting low-affinity clonotypes to their antigens in individuals who had a prior infection with common cold coronaviruses (17). Some of the TCRs, such as GV33 #7-8, #1, #57, GV34 #43, and #34-6, retained their response toward the variant peptides derived from OC43 and HKU1 at the higher concentration (100 nM), and all TCRs weakly recognize those from NL63 and 229E, consistent with previous findings (12, 15). These results indicated that QI9/A24-specific dominant TCRs in vaccinated individuals showed fine specificity for the SARS-CoV-2-derived QI9 peptide and lower cross-reactivity for other coronavirus-derived variant peptides.

**Table 3:** HLA bindig of variant peptides.

Name	Sequence	log Kd
QI9 (WT, SARS-CoV-2)	QYIKWPWYI	-6.80
SARS-CoV-1	--------V	-6.64
MERS	Y-N------	-6.96
NL63	N------WV	-6.05
229E	T------WV	-6.50
OC43	Y-V-----V	-6.64
HKU1	M-V-----V	-6.62
QI9-1A	A--------	-7.30
QI9-2A	-A-------	> -3
QI9-3A	--A------	-7.24
QI9-4A	---A-----	-7.43
QI9-5A	----A----	-7.41
QI9-6A	-----A---	-7.28
QI9-7A	------A--	-7.56
QI9-8A	-------A-	-7.65
QI9-9A	--------A	-5.95
pyrQI9	pyrQ--------	-7.00

To further investigate the cross-reactive potency of QI9/A24-specific TCRs, we examined TCR cross-reactivity toward alanine-substituted variant peptides of the SARS-CoV-2-derived QI9/A24 peptide at each position. An HLA binding assay revealed that QI9-2A and QI9-9A did not bind well to the HLA-A*24:02 molecule, consistent with the fact that position 2 and the C-terminus are known to be anchor residues for HLA-A*24:02 binding (**Table 3**) (18). All TCRs tested were tolerant toward the substitutions at the N-terminus and position 3, but not those at positions 5, 6, 7, and 8 (**Figure 1C**), and the TCR cross-reactivity toward the substitution at position 4 varied among TCRs. The TRAV19*01/TRBV20-1*01 clonotypes, but not others, retained their capacity to recognize the substitution. These TCR cross-reactivity profiles may explain why QI9/A24-specific TCRs retained some reactivity toward variant peptides from common cold coronaviruses such as OC43 and HKU1 but not NL63 and 229E, because they are tolerant of substitutions at the N-terminus and position 3, where substitutions are located (**Figures 1A, B**). Taken together, these data indicate that QI9/A24-specific TCRs retain their reactivity toward substitutions at the N-terminus and position 3.

### N-terminal pyroglutamation of the QI9/A24 peptide enhances proteasome resistance and TCR recognition

**Figure 2:**
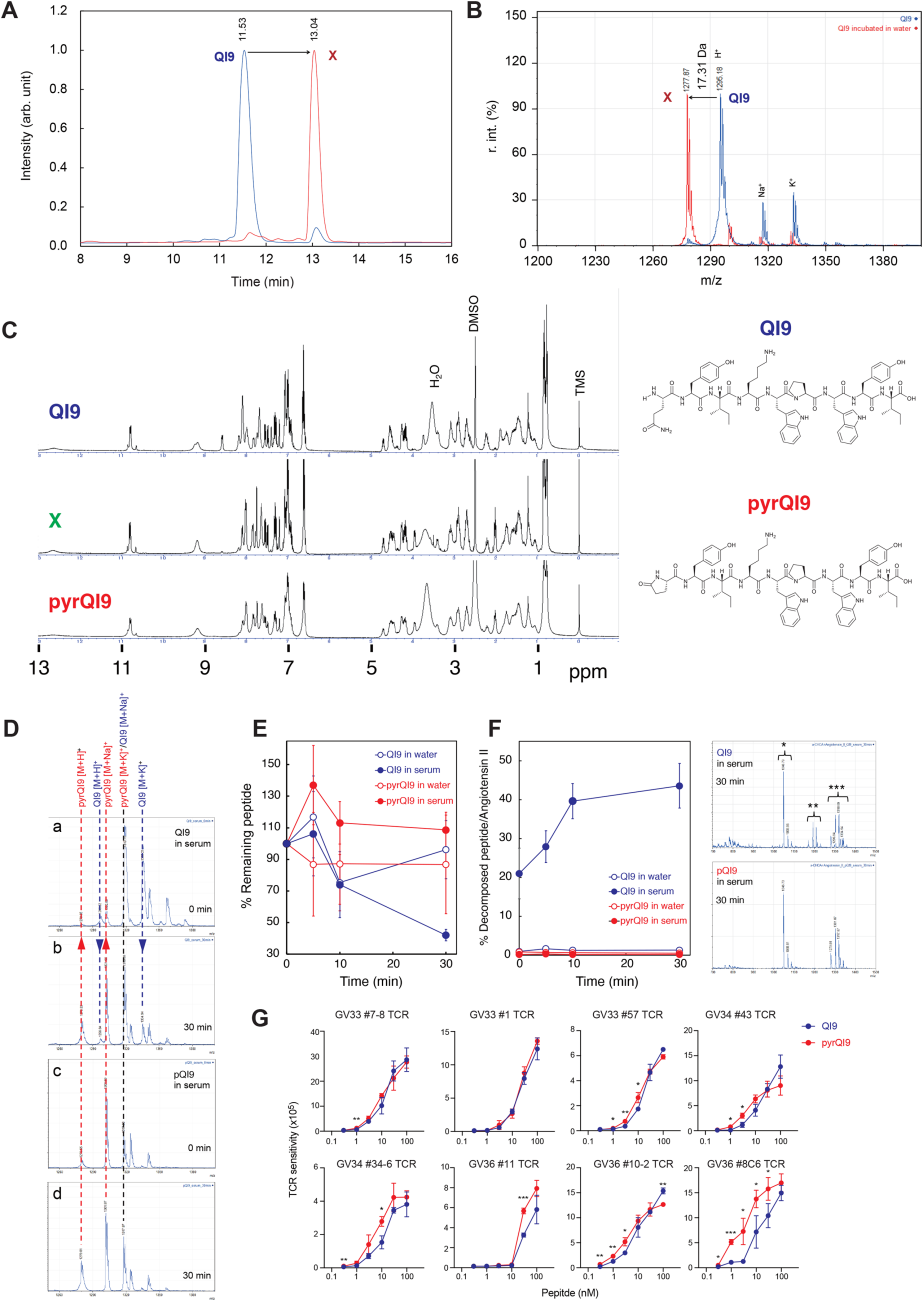
The N-terminal pyroglutamate QI9/A24 enhanced the recognition of QI9/A24-specific TCRs. **(A)**, HPLC chromatograms of the QI9 peptide at 1 day (blue solid lines) and after 17 days (red solid lines), and monitoring at 230 nm with a gradient of 10%-80% for 20 min (buffer A: 0.1% aqueous TFA and buffer B: acetonitrile). **(B)**, MALDI-TOF mass spectrum of the QI9 peptide. An α-CHCA matrix was used. The QI9 peptide; calcd. [M+H]+= 1,296.67 and obsd. [M+H]+= 1,295.18. MALDI-TOF mass spectrum of **(X)** An α-CHCA was used as a matrix. QI9; calcd. [M+H]+= 1,296.67 and obsd. [M+H]+= 1,277.87. **(C)** NMR spectra of the QI9 peptide, unknown sample X after incubating the QI9 peptide for 17 days, and pyrQI9 prepared from solid-phase peptide synthesis (SPPS) using pyroglutamic acid as one of the building blocks. **(D)**, MALDI-Tof mass spectra of QI9 after 0 min (a) and 30 min (b) in serum, and of pyrQI9 after 0 min (c) and 30 min (d) in serum. α-CHCA was used as the matrix. **(E)**, Plots of mass peak intensity (QI9 + pyrQI9) versus incubation time in each peptide solution (mean ± se, n = 3). Angiotensin II was used as the internal standard. **(F)**, Left panel: plots of mass peak intensity of a peptide with an N-terminal amino acid removed versus incubation time in each peptide solution (mean ± se, n = 3). Angiotensin II was used as the internal standard. Right panel: MALDI-Tof mass spectra of QI9 and pyrQI9 after 30 min of incubation in serum. α-CHCA was used as the matrix. “*” indicates the peak of Angiotensin II, “**” indicates the peak of a peptide from which one amino acid unit has been removed at the N-terminus, and “***” indicates the peak of the peptide of QI9 and/or pyrQI9. **(G)**, Peptide titration of TCR-transduced Jurkat cell lines toward the QI9 (blue lines) and pyrQI9 peptide (red lines). Sensitivity was evaluated by NFAT-luciferase reporter activity in TCR-transduced Jurkat cells. A statistically significant difference versus the QI9 peptide was determined by an unpaired two-tailed Student’s t-test (*p < 0.05, **p < 0.01, ***p < 0.001). The assay was performed in triplicate, and the means are shown with the SD. Data are representative of two or three independent experiments.

The QI9/A24 epitope is reported to be highly conserved across variants and immunodominant in vaccinated and convalescent individuals harboring the HLA-A*24:02 molecule (11, 12, 15). To assess the conservation of this epitope in recent circulating SARS-CoV-2 variants, we analyzed amino acid mutation frequency within the QI9/A24 epitope using 5,401,282 SARS-CoV-2 genome sequences (see Materials and Methods for details). The frequency of SARS-CoV-2 variants harboring amino acid mutations within the QI9/A24 epitope was less than 0.05% (**Supplementary Figure 1C**), indicating that this region is highly conserved. During the synthesis of the QI9/A24 peptide, we noticed that when the crude peptide was left in TFA/MeCN solution, the main peak decreased, and other peaks increased. Therefore, we dissolved the purified QI9/A24 in TFA/MeCN and left it at room temperature for 17 days. Subsequently, HPLC confirmed the presence of a new peak, indicating the production of a compound other than the QI9/A24 peptide. To identify this compound, we collected the solution of the newly generated peak and measured its mass using mass spectrometry. The results showed that this compound had a mass 17 Da lower than that of the QI9/A24 peptide. The N-terminal glutamine (Gln) of proteins and peptides is known to undergo pyroglutamate (19, 20). In this reaction, NH_3_ is released; the NH_3_ mass of 17 Da led us to consider that QI9/A24 has undergone pyroglutamate oxidation in solution this time (**Figures 2A, B**). ^1^H-NMR measurements confirmed that the resultant (X in **Figure 2C**) was N-terminally pyroglutamate QI9 peptide (pyrQI9) (**Figure 2C**). These data indicate that the conversion of the N-terminal glutamine residue may occur spontaneously in the SARS-CoV-2 spike-derived QI9/A24 peptide.

It is reported that modified peptides containing non-canonical amino acid exhibit improved *in vivo* stability due to their protease resistance (21–23). We therefore compared the stability of the QI9/A24 and pyrQI9 peptide in human serum and for long-term biostability and immunogenicity (**Figure 2D**). QI9/A24 was rapidly degraded in human serum within 30 min in contrast to the pyrQI9 peptide (**Figure 2E**). Moreover, QI9/A24 peptide breakdown products were readily detected in human serum, reaching a plateau within 10 min, unlike those of pyrQI9 (**Figure 2F**). These data indicate that the pyrQI9 peptide is more stable and immunogenic than the QI9/A24 peptide.

Given that QI9/A24-specific TCRs were tolerant of alanine substitution at the N-terminus (**Figure 1C**), we investigated whether the pyrQI9 peptide could be recognized by QI9/A24-specific TCRs. We first examined HLA binding using the HLA stabilization assay and found no difference in HLA binding between the QI9 and pyrQI9 peptides (**Table 3**). We next evaluated the sensitivity of the peptide using cell lines expressing TCRs. We found that the pyrQI9 peptide was recognized more efficiently by most of the QI9/A24 TCRs, except GV33 #1 TCR, especially at low concentrations (<10 nM) (**Figure 2G**). These results indicate that pyroglutamation of the N-terminus of the QI9 peptide enhances the sensitivity of the peptide by most of the QI9/A24-specific TCRs.

### Detection of CD8^+^ T cells cross-reactive for the QI9/A24 and pyrQI9 peptide in vaccinated and convalescent donors harboring HLA-A*24:02 *ex vivo*

**Figure 3:**
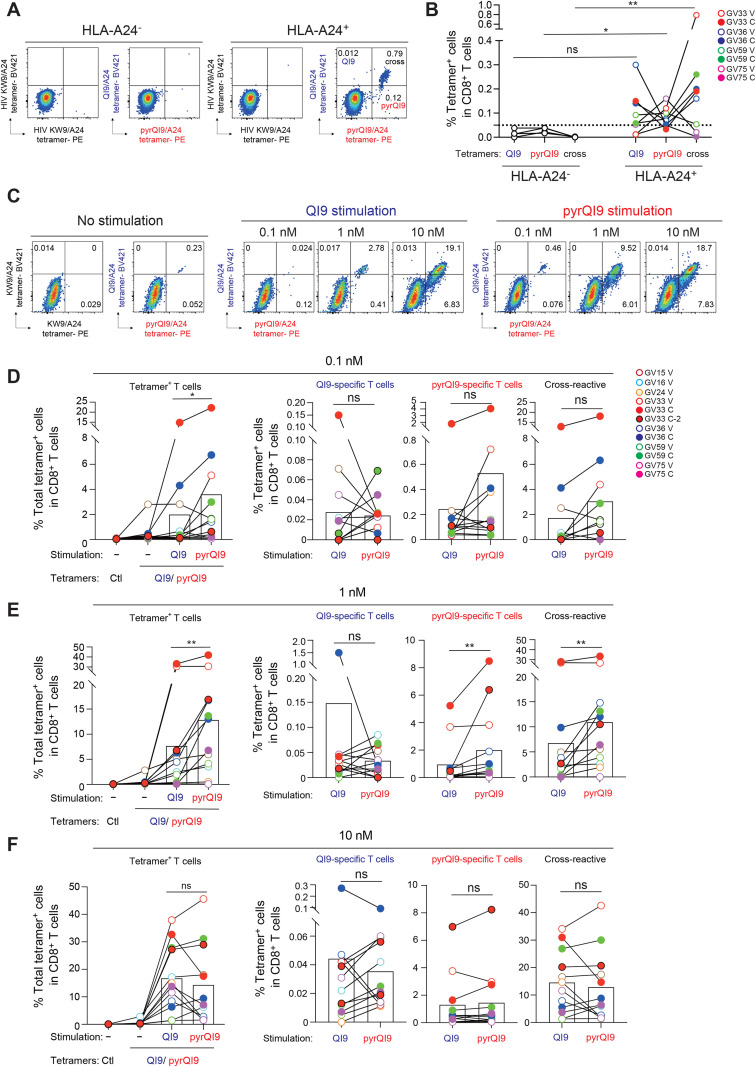
The N-terminal pyroglutamate QI9/A24 induces antigen-specific T cells effectively *in vitro.***(A)** Representative FACS plots showing HIV KW9 (Gag_28-36_: KYKLKHIVW)/A24 tetramer^+^ (control) or QI9/A24 and pyrQI9/A24 tetramer^+^ T cells of an HLA-A24-negative (left graphs, GV27V) and HLA-A24-positive (right graphs, GV33V) vaccinated donors *ex vivo*. An irrelevant tetramer was used as a negative control, and gating thresholds were defined accordingly. **(B)** The frequency of CD8^+^ T cells bound to QI9/A24 (blue), pyrQI9 (red) tetramers, or both (cross) in four vaccinated (V) and convalescent donors **(C)** is shown. **(C)** Representative FACS plots showing HIV KW9/A24 tetramer^+^ (control), QI9/A24 or pyrQI9/A24 tetramer^+^ T cells of an HLA-A24-positive convalescent donor (GV33 C-2) at 12 days after peptide stimulation of 0.1, 1, and 10 nM. An irrelevant tetramer was used as a negative control, and gating thresholds were defined accordingly. **(D–F)** The frequency of total tetramer^+^ CD8^+^ T cells that encompass QI9/A24, pyrQI9/A24, and both tetramer^+^ populations (left graph) and each tetramer^+^ T-cell subset (right graphs) at 0.1 nM **(D)**, 1 nM **(E)**, and 10 nM **(F)** of the QI9 and pyrQI9 peptide. **(D)** Statistically significant difference between the indicated peptides (*p = 0.0425) in total tetramer^+^ CD8^+^ T cells is determined by a two-tailed Wilcoxon matched-pair signed-rank test. **(E)** Statistically significant difference between the indicated peptides in total tetramer^+^ CD8^+^ T cells (**p = 0.0049), pyrQI9/A24 tetramer^+^ T cells (**p = 0.0024), and cross-reactive CD8^+^ T cells (**p = 0.0015) is determined by a two-tailed Wilcoxon matched-pairs signed rank test. ns, no statistical significance.

We stained PBMCs from vaccinated and convalescent donors with QI9/A24 and pyrQI9/A24 tetramers to investigate CD8^+^ T cells specific for the QI9/A24 and pyrQI9 peptide in vaccinated and convalescents, respectively (**Figure 3A**; **Supplementary Figure 1D**). We detected CD8^+^ T cells bound to both the QI9/A24 and pyrQI9/A24 tetramers (**Figure 3A**). Of note, the frequency of exclusive pyrQI9/A24 tetramer^+^ T-cell subsets and cross-reactive subsets was detected at high frequency in vaccinated and convalescent donors (**Figure 3B**). On the other hand, the QI9/A24 tetramer exclusively bound to CD8^+^ T cells in a vaccinated donor, GV36V, suggesting that different TCRs recognize the QI9/A24 and pyrQI9 peptides. These results indicate that there are three subsets of CD8^+^ T cells that exclusively recognize the QI9/A24 peptide, the pyrQI9 peptide, or both peptides, in vaccinated and convalescent donors *ex vivo*.

### An N-terminal pyroglutamated peptide induces antigen-specific CD8^+^ T cells effectively *in vitro*

To analyze the capacity of the N-terminal pyroglutamated pyrQI9 peptide to induce antigen-specific T cells, we stimulated PBMCs from seven vaccinated donors and four convalescent donors with the QI9/A24 or pyrQI9 peptides. After 12 days, proliferating T cells were evaluated for their binding to QI9/A24 and pyrQI9/A24 tetramers (**Figure 3C**). The percentages of total tetramer^+^ subsets, bound to the QI9/A24 tetramer, pyrQI9/A24 tetramer, and both tetramers, following stimulation with 0.1 and 1 nM of pyrQI9 peptide were significantly higher than those following stimulation with the QI9 peptide (**Figures 3D, E**, left). Of note, the frequency of pyrQI9/A24 tetramer-positive and double-positive T cells was significantly increased at 1 nM of the peptide (**Figure 3E**). Moreover, the average frequency of pyrQI9/A24 tetramer-positive and double-positive T cells was increased at 10 nM. However, there was no significant difference (**Figure 3F**). Our *in vitro* data indicate that the N-terminal pyroglutamate peptide can induce a higher frequency of antigen-specific T cells at lower concentrations than the QI9/A24 in vaccinated and convalescent donors carrying HLA-A*24:02.

### Immunization of N-terminal pyroglutamated peptide induces efficient antigen-specific CD8^+^ T cells *in vivo*

**Figure 4:**
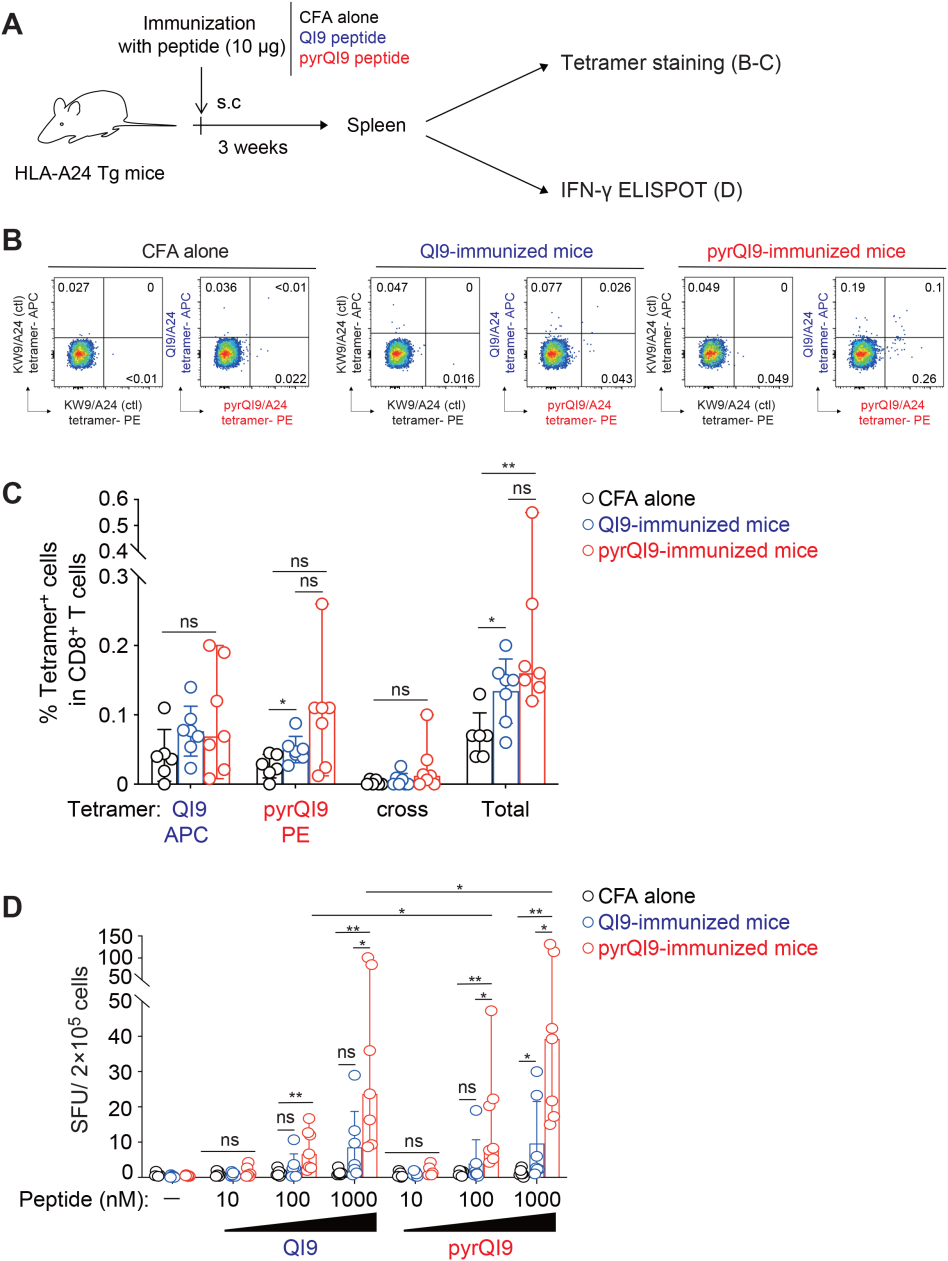
Immunization of HLA-A24 transgenic mice with the N-terminal pyroglutamated QI9 induces a potent antigen-specific T-cell response. **(A)** Schematic procedure of immunization and analysis of HLA-A24 transgenic mice immunized with peptides. **(B, C)** Representative FACS plots of tetramer double staining in CD8^+^ T cells from mice immunized without peptide (CFA alone) or with QI9 and pyrQI9 peptides and an irrelevant tetramer was used as a negative control, and gating thresholds were defined accordingly **(B)** and the frequency of total tetramer^+^ CD8^+^ T cells that encompass QI9/A24, pyrQI9/A24, and both tetramer^+^ populations (cross), and total tetramer^+^ T cells **(C)**. Statistically significant differences in the frequency of pyrQI9-reactive T-cell response in QI9-immunized mice (*p = 0.039 vs. CFA alone), total tetramer^+^ T cells in QI9-immunized mice, and pyrQI9-immunized mice (*p = 0.029 and **p = 0.0023 vs. CFA alone, respectively) are determined by the paired Wilcoxon signed-rank test. ns, no statistical significance. **(D)** Magnitude of T-cell response in stimulation of the QI9 or pyr QI9 peptides at 10, 100, and 1,000 nM in mice immunized with CFA alone (n=6), the QI9 (n=7), or the pyrQI9 peptide (n=7). When there was more than one spot in the negative control, positive responses were defined as more than twice that number. The assay was performed in duplicate, and the medians are shown with the range. A statistically significant difference between and within mice is determined by an unpaired Mann–Whitney t-test and the paired Wilcoxon signed-rank test, respectively. ns, no statistical significance. *p < 0.05 and **p < 0.01 were considered significant.

To investigate the ability of N-terminal pyroglutamate peptide to induce antigen-specific T cells, we immunized HLA-A24 transgenic mice with the pyrQI9 peptide and analyzed antigen-specific CD8^+^ T cells by tetramers and IFN-γ ELISpot (**Figure 4A**). The frequency of QI9/A24 and pyrQI9/A24 tetramer^+^ CD8^+^ T cells in mice immunized with the pyrQI9 peptide was higher than those immunized with the QI9/A24 peptide compared with those immunized with CFA alone (**Figures 4B, C**; **Supplementary Figure 1E**, **p = 0.0023 and *p = 0.0291 by Mann–Whitney test; versus CFA alone). Of note, we rarely detected both QI9/A24 and pyrQI9/A24 tetramer double-positive CD8^+^ T cells in mice immunized with the QI9/A24 and pyrQI9 peptides, unlike *ex vivo* tetramer staining of PBMCs from vaccinated and convalescent donors (**Figures 3A, B**), indicating the possibility of a TCR repertoire difference between mice and humans. Moreover, immunization of the pyrQI9 peptide induced higher IFN-γ production than that of the QI9/A24 peptide when the splenocytes were restimulated with QI9/A24 and pyrQI9 peptides at 100 and 1,000 nM (**Figure 4D**). In mice immunized with pyrQI9 peptide, the sensitivity of T cells in the pyrQI9 stimulation was also higher at 100 and 1,000 nM than that in the QI9/A24 stimulation (**Figure 4D**, *p = 0.0312 and *p = 0.0156 by Mann–Whitney test; versus the QI9/A24 stimulation). Collectively, these data indicate that the N-terminal pyroglutamate pyrQI9 peptide induces antigen-specific T cells more efficiently than the wild-type peptide by the peptide immunization *in vivo*.

### Vaccination with an N-terminal pyroglutamated peptide induces efficient antigen-specific CD8^+^ T cells that contribute to the immune response against SARS-CoV-2 challenge

**Figure 5:**
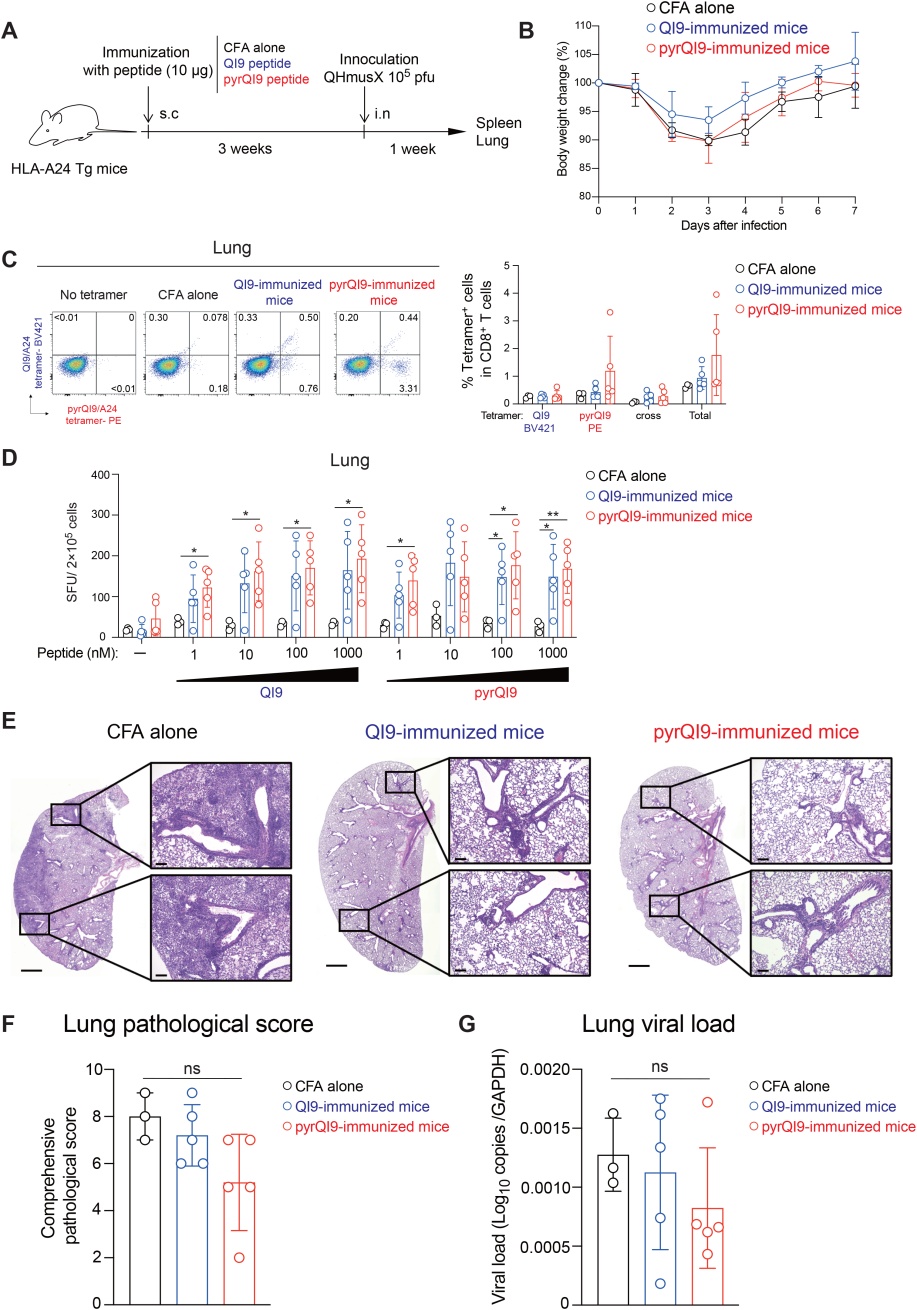
N-terminal pyroglutamated peptide induces efficient antigen-specific CD8^+^ T cells from lung against challenge with SARS-CoV-2 virus. **(A)** Schematic procedure of immunization and infection of HLA-A24 transgenic mice with peptides. **(B)** Body weight changes among the groups after inoculation. **(C)** Representative FACS plots of tetramer double staining in CD8^+^ T cells of lungs from mice immunized without peptide (CFA alone, N = 3) or with the QI9 (n=5) and pyrQI9 peptides (n=5) and the frequency of total tetramer^+^ CD8^+^ T cells that encompass QI9/A24, pyrQI9/A24, and both tetramer^+^ populations (cross), and total tetramer^+^ T cells. **(D)** Magnitude of T-cell response from lungs to the QI9 or pyrQI9 peptides in mice immunized with CFA alone (n=3), the QI9 (n=5), or the pyrQI9 peptide (n=5). When there was more than one spot in the negative control, positive responses were defined as more than twice that number. The medians are shown with the range. Statistical significance between groups was determined by an unpaired Mann–Whitney t-test and a paired Wilcoxon signed-rank test, respectively. ns, no statistical significance. *p < 0.05 and **p < 0.01 were considered significant. **(E)** Representative histopathological analysis among the groups after inoculation. **(F)** Lung pathology was evaluated by comprehensive pathological score. **(G)** SARS-CoV-2 viral load was measured by quantitative PCR using nucleocapsid gene-specific primers, and gene expression levels were normalized to *Gapdh*.

To further investigate the effect of immunization of N-terminal pyroglutamated peptide on viral infection, we immunized mice with the QI9/A24 and pyrQI9 peptide and performed intranasal challenge experiments with SARS-CoV-2 virus (QHmusX) (**Figure 5A**) (24). There was no difference in the body weight changes among the groups after inoculation (**Figure 5B**). The frequency of QI9/A24 and pyrQI9/A24 tetramer^+^ CD8^+^ T cells from splenocytes and lungs in mice immunized with the pyrQI9 peptide, QI9/A24 peptide, or CFA alone was not altered (**Figure 5C**; **Supplementary Figures 1F, G**, **2A**). Although the number of IFN-γ-producing cells in stimulation with the QI9/A24 and pyrQI9 peptide at 100 nM was significantly higher than that with CFA alone in spleen (**Supplementary Figure 2B**, *p = 0.0297 and 0.0445 by unpaired two-tailed Student’s t-test; versus CFA alone, respectively). Of note, in the lung, the number of IFN-γ-producing cells in pyrQI9-immunized mice, but not QI9/A24-immunized mice, was significantly higher than that in mice with CFA alone in stimulation with the QI9/A24 peptide (**Figures 5D**, left). Moreover, a similar tendency was observed in stimulation with the pyrQI9 peptide (**Figures 5D**, right). Histopathological analysis revealed that inflammatory features, including immune cell accumulation and alveolar epithelial thickening, were less pronounced in QI9/A24- or pyrQI9-immunized mice than in control mice (**Figure 5E**). Additionally, bronchus-associated lymphoid tissue (BALT) formation was enhanced in QI9/A24- or pyrQI9-immunized mice compared with control mice (**Figure 5E**). Histopathological scoring revealed that the average of pyrQI9-immunized mice tended to be lower than QI9-immunized mice and control mice, whereas there was no statistical difference (**Figure 5F**). Similar tendency was observed in the viral load in the lung (**Figure 5G**). However, there was no statistical difference in mRNA expression of inflammatory cytokines such as IFN-γ, TNF, CXCL10, and IL-6 between QI9- and pyrQI9-immunized mice (**Supplementary Figure 2C**). Taken together, these data indicate that immunization with the N-terminally modified pyrQI9 peptide induces potent antiviral T-cell responses coupled with reduced lung pathology following SARS-CoV-2 infection.

## Discussion

In this study, we characterized HLA-A*24:02-restricted T cells specific for an immunodominant QI9/A24 epitope that is highly conserved in vaccinated and convalescent individuals. QI9/A24-specific TCRs are composed of diverse TCR clonotypes that are cross-reactive to the variant peptides derived from other coronaviruses with amino acid substitutions at the N-terminus and position 3. At the N-terminus of the QI9/A24 peptide, which is one of the tolerable positions, we identified the pyroglutamated modification of the QI9/A24 peptide and demonstrated that the N-terminal pyroglutamate (pyrQI9) peptide with increased protease resistance induced antigen-specific T cells effectively *in vitro* and *in vivo*. These data indicate that modification of the N-terminus at a tolerable site of a peptide epitope can positively impact vaccination against viral infection.

Spontaneous modification of the N-terminal glutamine of a peptide epitope by pyroglutamate has been reported previously, but the effect on T-cell recognition varied (19, 20, 25). For example, the modification at the N-terminus of Rat MHC class II RT1.B^L^-restricted guinea pig myelin basic protein-derived peptide (gpMBP_72–85_: QKSQRSQDENPV) reduced the binding of MHC class II and subsequently its recognition by T cells (20). Moreover, the N-terminal pyroglutamation of HLA-A2-restricted melanoma peptide (p68_315–323_: QIVDVCHDV) also interferes with the binding of the peptide to HLA-A2 and decreases CTL recognition (19). On the other hand, the N-terminal pyroglutamation of MHC L^d^-restricted QLSPFPFDL was efficiently recognized by 2C T cells via enhanced MHC binding activity (25). Here, we showed that the N-terminal pyroglutamate of SARS-CoV-2-derived immunodominant QI9/A24 peptide enhanced TCR recognition. To our knowledge, this is the first report demonstrating the positive effect of the N-terminal pyroglutamation of a T-cell epitope on antiviral T-cell responses.

Several limitations of the present *in vivo* experiments should be noted. Firstly, this strategy, which involves N-terminal pyroglutamation, requires an N-terminal glutamine in the epitope peptide, and its effect on HLA binding or T-cell recognition depends on the amino acid sequence. Mechanistically, we speculated that the N-terminus modification of pyroglutamate indirectly affects the overall structure of the peptide presented by the HLA-A*24:02 molecule. In support of this, structural modeling predicted that the N-terminal pyroglutamate residue interacts with an additional residue, Try183 of HLA-A*24:02 (**Supplementary Figure 2D**). This additional contact may explain the structural fluctuation of Trp5 and Tyr8 of the pyrQI9 peptide (**Supplementary Figure 2E**) and the close distance between Trp7 and Trp8 in the pyrQI9 peptide (**Supplementary Figure 2F**), which may contribute to enhance the sensitivity of QI9/A24-specific TCRs. Future crystallographic studies of the TCR-peptide/HLA complex will determine the mechanism by which N-terminal pyroglutamatation enhances TCR recognition, providing rational insights for the broader application of N-terminal modification with non-canonical amino acids to other immunodominant epitopes. Secondly, the *in vivo* experiments were conducted using modest group sizes and a single peptide dose, which represents a limitation of the current study. Although these conditions were sufficient to demonstrate consistent trends following viral challenge, future studies incorporating larger cohorts and dose-ranging analyses will be important to further strengthen the interpretation of these findings.

It is known that TCRs can recognize many different antigenic peptides in the context of a single HLA molecule (26). Despite the intrinsic degeneracy of TCR recognition, αβ TCRs typically bind to peptide-HLA complexes in a diagonal orientation over the center of the peptide, and the residues at the N-terminus of the peptide generally are not involved in the TCR contacts (27). In this study, we demonstrated that modifying the N-terminal residue of the QI9/A24 peptide improved TCR recognition. Considering universal vaccine designs for another epitope peptide, targeting the modification of the N-terminal residue might be an effective strategy to enhance T-cell responses (28).

The conversion of N-terminal glutamine to pyroglutamine, catalyzed by glutamine cyclase (QC), is an important posttranslational modification involving the cyclization of the N-terminal glutamine or glutamate residues of various peptides and proteins to form pyroglutamate (29, 30). However, it is unclear whether QC converts the N-terminal glutamine of the QI9/A24 peptide to its cyclized form under physiological conditions, such as during mRNA vaccination or infection. Here, *ex vivo* staining of PBMCs with HLA tetramers revealed that the frequency of T cells binding the pyrQI9/A24 tetramer was higher than for the QI9/A24 tetramer and both tetramers in a vaccinated donor, GV75V, suggesting the presence of pyrQI9-specific T cells following mRNA vaccination. This may support the idea that the pyrQI9 peptide is presented by HLA-A*24:02 molecules on spike-expressing antigen-presenting cells or virally-infected cells. Further TCR clonotype analysis of CD8^+^T cells specific for QI9/A24 or pyrQI9, and of double-positive T cells, will reveal the functional differences in T cells induced by the QI9/A24 or pyrQI9 peptide.

Antigenic peptides with non-canonical amino acid are attractive candidates for peptide-based vaccines. A modified Flu epitope peptide composed of D-amino acids primed protective immune responses in a humanized mouse model of influenza virus infection (21). This peptide improved not only the TCR-pMHC interaction but also *in vivo* stability, due to its protease resistance, compared with the wild-type peptide (21–23). The conversion of N-terminal glutamine to pyroglutamine is equivalent to introducing a non-canonical amino acid in the peptide. Considering that this conversion may occur under physiological conditions, introducing preexisting non-canonical amino acid into antigenic peptides may be safe and provide better insights for developing vaccine design.

## Methods

### Ethics statement

For the use of human specimens, all protocols involving human subjects recruited at Kumamoto University were reviewed and approved by the Institutional Review Boards of Kumamoto University (approval numbers 461 and 477). All human subjects provided written informed consent. For the use of mice, this study was approved by the Committee of Ethics on Animal Experiments in the Joint Research Center for Human Retrovirus Infection of Kumamoto University (approval numbers A2024–067 and 5-034) and the Nara Medical University (approval number 13516). Experiments were carried out under the control of the Guidelines for Animal Experiments. Mice were maintained under a 12-h dark/light cycle and constant conditions of temperature (18 °C–23 °C) and humidity (40%–60%). Transgenic mice, HHD (a kind gift from Dr. F. A. Lemonnier, Pasteur Institute, Paris, France), express a transgenic HLA-A*24:02 monochain in which human beta-2 microglobulin (β2m) is covalently linked to a chimeric heavy chain composed of HLA-A*24:02 (α1 and α2, domains) and H-2Db (α3, transmembrane, and cytoplasmic domains) (31). 8- to 11-week-old mice were used for all experiments. Following anesthetic induction by the inhalation of isoflurane (4-5%, Wako, Cat# 099-6571) or the intraperitoneal administration or a combination of medetomidine (0.75 mg/kg, Nippon Zenyaku Kogyo), midazolam (4 mg/kg, Maruishi Pharmaceutical), and butorphanol (5 mg/kg, Meiji Animal Health), euthanasia was performed by exsanguination or cervical dislocation.

### Collection of human PBMCs

Human PBMCs were obtained from seven HLA-A*24:02-positive BNT162b2-vaccinated donors (median age: 32, range: 23-40, 86% male), four HLA-A*24:02-negative BNT162b2-vaccinated donors (median age: 27, range: 24-34, 25% male), and five convalescent donors (median age: 39, range: 38-40, 100% male) (**Table 1**). PBMCs were purified by a density gradient centrifugation using Ficoll-Paque Plus (GE Healthcare Life Sciences, Cat# 17-1440-03) and stored in liquid nitrogen until further use.

### Peptide synthesis

We synthesized peptides using conventional 9- fluorenylmethyloxycarbonyl (Fmoc)-based solid-phase peptide synthesis. Piperidine was used for the deprotection, and trifluoroacetic acid (TFA) (WATANABE CHEMICAL, Cat# A00026) for the cleavage. We used Fmoc-Ile-Wang Resin as the solid support. All experimental processes were performed at room temperature. First, the resin was swollen in a 1:1 mixture of dimethylformamide (DMF) (Nacalai Tesque, Cat# 04096-21) and dichloromethane (DCM) (Wako, Cat# 132-02456), and the Fmoc group was deprotected using 20% piperidine in DMF. The coupling, deprotection, and washing processes were then repeated to complete the peptide to the desired sequence. For coupling, the amino acids were dissolved in DMF with a coupling cocktail containing O-(1H-benzotriazol-1-yl)-N,N,N,N′-tetramethyluronium hexafluorophosphate and N-methylmorpholine (Wako, Cat# 132-06873). After washing the resin with dichloromethane, cleavage cocktail [TFA/triisopropylsilane/water (95/2.5/2.5 v/v/v)] was added and incubated for 1.5 h at room temperature to cleave the peptide from the resin and further deprotect the protecting groups on the side chains. The crude peptide was precipitated in diethyl ether and further washed with diethyl ether until a neutral pH was reached. The peptides were purified using reverse-phase high-pressure liquid chromatography (RP-HPLC) on a C18 preparative column (Cadenza 5CD-C18; Imtakt, Kyoto, Japan). Final product identification was performed using matrix-assisted laser desorption/ionization-time-of-flight (MALDI-ToF) mass spectrometry (Shimadzu AXIMA Confidence, Shimadzu, Kyoto, Japan) and HPLC on a C18 analytical column [Cadenza CD-C18 Column (250 × 20 mm, 5 μm particle diameter; Imtakt, Kyoto, Japan)]. The peptides with > 95% purity were used in this study.

### NMR measurement

^1^H-NMR measurements of the peptides were performed with a JNM-AL 400 (JEOL, Tokyo, Japan; 400 MHz). DMSO-d_6_ was used as the solvent, and the measurement temperature was 25 °C. For sample X in **Figure 2**, the QI9/A24 peptide was incubated in 0.1% aqueous TFA/acetonitrile (= 3/1 v/v) at room temperature for 17 days. After that, it was analyzed by HPLC, and its main component was fractionated by preparative HPLC. The component was lyophilized, and the obtained solid was measured by NMR. The pyrQI9 peptide was prepared by SPPS in the same way as the QI9/A24 peptide, and pyroglutamic acid was used as the building block of the N-terminus instead of Fmoc-Gln(Trt)-OH (Q). The same procedure was used to purify the pyrQI9 peptide as for the QI9/A24 peptide, and the solid was analyzed by NMR.

### Protease stability of peptides

Protease stability of the QI9/A24 and pyrQI9 peptides was assessed following a modified protocol as previously described (21). Briefly, human serum from AB plasma (Sigma-Aldrich, Cat# 4522) was centrifuged at 200,000 × g for 10 min to remove the lipid components. The supernatant was diluted to 25% (v/v) with Milli-Q water and incubated at 37 °C for 15 min. The QI9/A24 and pyrQI9 were diluted in 25% serum to a final concentration of 50 μg/mL. Control samples were prepared by diluting peptides to the same concentration in Milli-Q water. All samples were incubated at 37 °C, and aliquots of 100 μL were collected at 0, 5, 10, and 30 min and mixed with an equal volume of acetonitrile (FUJIFILM Wako Pure Chemical Corporation, Osaka, Japan, Cat#: 015-08633) and centrifuged for 1 min at 1,500 × g. The supernatant was analyzed by MALDI-Tof mass spectrometry (Shimadzu, MALDI-7090). Stability was calculated as the sum of the intensities of the peaks corresponding to Angiotensin II and to the full-length substrate peptides (derived from QI9 or pyrQI9, including H^+^-, Na^+^-, and K^+^-adducts), and the ratio of the sum of the peak intensity of QI9 (or pyrQI9) to that of Angiotensin II (used as the internal standard) at time 0 was determined. The same ratio was then calculated at 5, 10, and 30 min. Finally, the ratio at time 0 was defined as 100%, and the ratios at subsequent time points were expressed as percentages to indicate the residual peptide levels.

### Cell culture

A549/A2402 cells, the A549 cells stably expressing HLA-A*24:02-IRES-GFP, were generated by plasmid DNA Transfection and were maintained in Ham’s-F12 (Wako, Cat# 080-08565) containing 10% fetal bovine serum (FBS). TCR-deficient Jurkat cells expressing the luciferase gene (JurkatΔ-Luc) were maintained in RPMI 1640 medium (Thermo Fisher Scientific, Cat# 11875101) containing 10% FBS.

### The peptide-dependent stabilization assay

The HLA-binding of peptide was analyzed as previously described (9, 32). Briefly, TAP-deficient C1R-A24 cells were incubated at 26 °C overnight. A total of 1 × 10^5^ cells was incubated with 1 μM β2-microglobulin (β2m) and graded concentrations of peptides. Cells were incubated at 26 °C for 1 h and then at 37 °C for 4 h. At the end of the incubation, unbound peptides were removed, and cells were stained with FITC-labeled Bw4-specific mAb 17A12 (20 mg/mL) provided by Dr. Ulrich Hämmerling at Memorial Sloan Kettering Cancer Center and analyzed by FACScan (BD, San Jose, CA, USA). The mean fluorescence intensity (MFI) was calculated using CellQuest, and the mean values from duplicate samples were presented. Peptide binding is normalized by high-binder peptide (TYLPTNASL) and low-binder peptide (RVWESATPL) in each experiment.

### Analysis of SARS-CoV-2 genomic variation

To investigate genomic mutations in SARS-CoV-2, we downloaded 17,495,450 genome sequences from the GISAID database as of November 16, 2025 (33). After further filtering for sequences that 1) were derived from human hosts, 2) exceeded 29,000 nucleotides in length, or 3) contained ambiguous or degenerate bases, a total of 5,401,282 genome sequences were retained for mutation analysis. The GISAID IDs used are accessible at https://doi.org/10.55876/gis8.251126nq. For each sequence, SGV-caller was used to identify genomic mutations at nucleotide and amino acid levels (34).

### Tetramer staining

SARS-CoV-2-derived peptides-loaded MHC class I tetramers were generated by QuickSwitch Quant HLA-A*24:02 Tetramer Kit-PE, -APC, and -BV421 according to the manufacturer’s protocol. PBMCs were stained with tetramers for 30 min. After tetramer staining, surface staining was performed with the following antibodies: For human, CD3 BV421 (UCHT1, 1/100 dilution; BioLegend, Cat# 300434), CD3 PE (OKT3, 1/200 dilution; BioLegend, Cat# 317308), CD3 AF532 (UCHT1, 1/25 dilution; eBioscience, Cat# 58-0038-42), CD8 Pacific Blue (HIT8a, 1/100 dilution; BioLegend, Cat# 300928), CD14 PerCP/Cy5.5 (HCD14, 1/100 dilution; BioLegend, Cat#325622), CD4 BV750 (SK3, 1/100 dilution; BioLegend, Cat# 344644). For mice, CD3 PE (145-2C11, 1/50 dilution; BioLegend, Cat# 100308), CD3 APC (145-2C11, 1/200 dilution; BioLegend, Cat# 100312), CD4 BV750 (GK1.5, 1/200 dilution; BioLegend, Cat# 100467), CD4 AF700 (GK1.5, 1/100 dilution; BioLegend, Cat# 100430), CD8a BV570 (53-6.7, 1/200 dilution; BioLegend, Cat# 100740), CD8a AF647 (KT15, 1/100 dilution; MBL, Cat# K0227-A64), CD19 BV510 (1D3/CD19, 1/100 dilution; BioLegend, Cat# 152423), CD45 PerCP (30-F11, 1/100 dilution; BioLegend, Cat# 103130), CD45R/B220 APC Fire750 (RA3-6B2, 1/200 dilution; BioLegend, Cat# 103260), anti-mouse TCR β BV785 (H57-597, 1/200 dilution; BioLegend, Cat# 109249), TCR β APC-Cy7 (H57-597, 1/100 dilution; BioLegend, Cat# 109219), CD62L FITC (MEL-14, 1/100 dilution; BioLegend, Cat# 104406), and CD44 PerCP/Cy5.5 (IM7, 1/100 dilution; BioLegend, Cat# 103032) was performed. Dead cells were stained with 7-aminoactinomycin D (1/50 dilution) or Zombie-NIR (1/200 dilution; BioLegend, Cat# 423105). After incubation for 20 min, the cells were fixed with 1% paraformaldehyde (Nacalai Tesque, Cat# 09154-85), and the levels of tetramer^+^CD8^+^ T cells were analyzed by flow cytometry using a Cytek Northern Lights (Cytek Japan), followed by analysis using FlowJo v10 software (BD Biosciences).

### *In vitro* stimulation of PBMCs with the peptides

Human PBMCs were pulsed with 0.1, 1, and 10 nM of the QI9/A24 (QYIKWPWYI, residues 1,208-1,216 of the SARS-CoV-2 spike protein) and the N-terminal pyroglutamated pyrQI9 peptide maintained in RPMI 1640 medium (Thermo Fisher Scientific, Cat# 11875101) containing 10% FBS and 30 U/mL recombinant human IL-2 (PeproTech, Cat# 200-02) for 12 days. The HLA-A*24:02-restricted KW9 (HIV Gag_28-36_: KYKLKHIVW) peptide was used as a negative control. The *in vitro* expanded CD8^+^ T cells were stained with HLA-A24 tetramers. The cells were washed and stained with tetramers for 30 min. After tetramer staining, surface staining was performed with the following antibodies: CD3 BV421 (UCHT1, 1/100 dilution; BioLegend, Cat# 300434), CD3 PE (OKT3, 1/200 dilution; BioLegend, Cat# 317308), CD3 AF532 (UCHT1, 1/25 dilution; eBioscience, Cat# 58-0038-42), CD8 Pacific Blue (HIT8a, 1/100 dilution; BioLegend, Cat# 300928), CD14 PerCP/Cy5.5 (HCD14, 1/100 dilution; BioLegend, Cat#325622), CD4 BV750 (SK3, 1/100 dilution; BioLegend, Cat# 344644). Dead cells were stained with 7-aminoactinomycin D (1/50 dilution). After incubation for 20 min, the cells were fixed with 1% paraformaldehyde (Nacalai Tesque, Cat# 09154-85), and the levels of tetramer^+^CD8^+^ T cells were analyzed by flow cytometry using a Cytek Northern Lights (Cytek Japan), followed by analysis using FlowJo v10 software (BD Biosciences).

### Jurkat reporter cell (JurkatΔ-Luc) for functional analysis of TCRs

A DNA fragment of NFAT-RE-Luc2P-SV40 pro-HygroR was amplified from pGL4.3 (Promega) by PCR. The DNA fragment was cloned into the Stu I/Sal I site of PiggyBac vector PB530A-2 (SBI) by the Gibson assembly method. The resultant vector [PB_NFAT-RE-Luc2P-SV40 pro-HygroR] was electroporated into endogenous TCR knocked-out Jurkat cells using the transposase expression vector, PB200PA-1 (SBI). To select the Jurkat reporter cell (JurkatΔ-Luc) integrated with NFAT-RE-Luc2P-SV40 pro-HygroR, Hygromycin-B selection was performed at 500 μg/mL concentration for 14 days.

### TCR cDNA amplification from single T cells and construction of TCR expression vector

The cryopreserved PBMCs were stained with NF9/A24 and QI9/A24 tetramers, anti-CD8 mAb (RPA-T8; BioLegend), and 7-amino-actinomycin D (7-AAD), and then tetramer^+^CD8^+^7-AAD^−^ cells were sorted into 96-well plates (NIPPON Genetics, Cat# 4ti-0770/C) by using an FACSAria II (BD Biosciences). TCRα and TCRβ cDNA pairs were amplified from single T cells by a one-step multiplex RT-PCR method described in our previous study (35). The DNA sequences of the PCR products were then analyzed by direct sequencing, and the TCR repertoire was analyzed using IMGT/V-QUEST (https://www.imgt.org/IMGT_vquest/vquest). The amplified TCRα and TCRβ cDNA fragments were fused to the missing constant regions and linked to the blasticidin S resistance (BlaR) gene using the Gibson assembly method with P2A ribosomal skipping sequences. Resultant TCRβ-P2A-TCRα-P2A-BlaR DNA was cloned into the PiggyBac vector (SBI, Cat# PB530A-2) by the Gibson assembly method.

### TCR sensitivity assay

The plasmid PB TCR-P2A-BlaR was electroporated into JurkatΔ-Luc with the transposase vector (SBI, Cat# PB200PA-1) using the Neon^®^ Transfection System (Thermo Fisher Scientific) under the conditions 1,200 v, 5 ms, and five pulses. After 48 h, JurkatΔ-Luc cells stably expressing TCRs were selected with RPMI medium containing 10 μg/mL of blasticidin-S for 10–14 days. These cells were cocultured with A549-ACE2-A2402 cells expressing each spike protein at an E:T ratio of 2:1 and incubated in RPMI 1640 medium (Thermo Fisher Scientific, Cat# 11875101) containing 10% FBS at 37 °C for 6 h. The mixture was measured for luciferase production using a luminescent substrate (Promega, Cat#E2510) by a CentroXS3 plate reader (Berthold Technologies).

### Immunization

Each peptide was dissolved in PBS and emulsified with an equal volume of Complete Freund’s adjuvant (CFA) (BD Difco, code: DF0638-60-7). HLA-A24 transgenic mice were immunized intradermally with 100 μL of the emulsion containing 10 μg of either peptide. Control mice were given CFA emulsion containing no peptide. HLA-A24 transgenic mice were immunized with CFA alone (n = 6, 66.7% male, 10–11 weeks old), the QI9/A24 peptide (n = 7, 71.4% male, 8–11 weeks old), or the pyrQI9 peptide (n = 7, 57.1% male, 8–11 weeks old).

### IFN-γ ELISpot

An *ex vivo* IFN-γ ELISpot assay was performed using the following antibodies and reagents: anti-mouse IFN-γ mAb AN18, purified (Mabtech, Code: 3321-3-1000), anti-mouse IFN-γ mAb R4-6A2, biotinylated (Mabtech, Code: 3321-6-1000), streptavidin-ALP (Mabtech, Code:3310-8-1000), 25×AP Color Development Buffer (Bio-Rad), AP Conjugated Substrate Kit (Bio-Rad, Cat# 1706432) according to the manufacturer’s protocol. Briefly, after washing a MultiScreen 96-well plate with sterilized PBS and blocked with RPMI 1640 medium (Thermo Fisher Scientific, Cat# 11875101) containing 10% FBS, 2×10^5^ splenocytes from HLA-A24 transgenic mice immunized with CFA alone, the QI9/A24 peptide, and the pyrQI9 peptide 3 weeks after immunization per well were pulsed with the QI9/A24 or pyrQI9 peptide and cultured for 20 h. The responses were defined as the number of spot-forming cells (SFC)/2×10^5^ calculated by ImmunoSpot (Cellular Technology Limited). When there was more than one spot in the negative control, the responses were defined as more than twice the background.

### SARS-CoV-2 infection

A mouse model of SARS-CoV-2 infection was prepared using a mouse-adapted SARS-CoV-2 strain (QHmusX), kindly provided by the National Institute of Infectious Diseases, Japan (24). HLA-A24 transgenic mice were immunized with CFA alone (n = 3, 100% male, 8–10 weeks old), the QI9/A24 peptide (n = 5, 20% male, 8–10 weeks old), or the pyrQI9 peptide (n = 5, 20% male, 8–10 weeks old). 21 days after immunization, mice were intranasally inoculated with 1×10^5^ PFU of SARS-CoV-2. Mice were weighed daily, and they were euthanized for analysis 7 days after infection. The right lung lobes were minced and dissociated using a Lung Dissociation Kit (Miltenyi Biotec, Cat# 130-095-927) with a gentleMACS Octo Dissociator (Miltenyi Biotec) according to the manufacturer’s instructions. Then, debris was removed by a 25% Percoll gradient (Cytiva, Cat# 117544502). Lung cells and splenocytes were treated with Red Blood Cell Lysis Buffer (Sigma-Aldrich, Cat# R7757) and subsequently used for tetramer staining and IFN-γ ELISpot assay, as described above. The left lung lobes were fixed in 4% paraformaldehyde and embedded in paraffin. Sections were sliced to a thickness of 1–2 μm and stained with hematoxylin and eosin (Sakura Finetek Japan Co). All images of the sections were obtained with a BioZero BZ-X810 microscope (KEYENCE, Osaka, Japan). Lung pathology was evaluated based on three criteria: (a) alveolar septal thickening and consolidation; (b) hemorrhage, exudation, pulmonary edema, and mucus; and (c) recruitment and infiltration of inflammatory cells, as previously described (36). Each category was scored by severity: 0 (none), 1 (mild), 2 (moderate), 3 (severe), and 4 (very severe). The total pathological score was calculated as the sum of these individual scores. Blinded histological assessment was performed by a trained pathologist.

### Measurement of viral RNA

Total RNA was extracted from homogenized right lung lobes from infected mice using an RNeasy Mini kit (Qiagen, Cat# 74104) and then reverse-transcribed with a High-Capacity cDNA Reverse Transcription Kit (Thermo Fisher Scientific, Cat# 4368814). Quantitative PCR was performed on a QuantStudio 3 system (Thermo Fisher Scientific) using the following TaqMan Gene Expression Assays: *Cxcl10* (Mm00445235), *Ifng* (Mm01168134), *Il6* (Mm00446190), and *Tnf* (Mm00443258). To determine the SARS-CoV-2 viral load, nucleocapsid gene was targeted using the NIID-N2 set (National Institute of Infectious Diseases, Japan), which consists of a forward primer (5′-AAATTTTGGGGACCAGGAAC-3′) and reverse primer (5′-TGGCAGCTGTGTAGGTCAAC-3′) and TaqMan probe (FAM-ATGTCGCGCATTGGCATGGA-BHQ). As the target sequence is located within the N gene, this assay detects both genomic RNA and subgenomic nucleocapsid mRNA. Gene expression levels were normalized to *Gapdh* (Thermo Fisher Scientific, Cat# Mm99999915) expression as an internal control.

### Structural modeling and simulation of the peptide/HLA-A*24:02 molecule

X-ray crystal structures of the QI9/HLA complex were obtained from the PDB (Protein Data Bank) (PDB IDs: 8HN4). We also used the AlphaFold Server (37) to predict the structures of the QI9/HLA and pyrQI9/HLA complexes. The predicted model structures were used for MD simulations of the QI9/HLA complex. MOE 2020.09 was used to generate the initial structure for the MD simulation. Mutations were introduced using Builder, and structural modifications were made using Structure Preparation. Hydrogen atoms were added using Protonate 3D, and the structure was optimized using the Amber10:EHT force field in Energy Minimize.

### MD simulation

The force field was added using AmberTools22. Amber ff14SB force fields were used for proteins and ions, and TIP3P for the solvent model. Water molecules were placed in a 20-Å periodic boundary box around the protein and neutralized with Na+. Note that due to the large molecular weight of the pHLA complex, water molecules were placed in periodic boundary boxes of 148 Å per side. The MD simulation and its analysis were performed using GROMACS 2021.5. The particle mesh Ewald (PME) method was used for the electrostatic interactions with a cutoff radius of 12 Å. Similarly, the cutoff radius for van der Waals interactions was set to 12 Å. The LINKS algorithm was used to fix the bond lengths. The Steepest Descent method was used for energy minimization. The V-scale method was used to control the equilibration temperature to 310.15 K, and the C-scale method was used to maintain the pressure at 1,000 hPa. The energy of the system was first minimized to 10.0 kJ/mol/nm, followed by 200 ps of heating (NVT) and 800 ps of density relaxation (NPT). These equilibration processes were performed with a gradual relaxation of the atomic restraints. Finally, 50-ns simulations were run three times for each structure. For each analysis method, the root mean square deviation (RMSD) was calculated by acquiring 501 frames at 0.1-ns intervals, starting at 0 ns, and 401 frames at 0.1-ns intervals, starting at 10 ns, for the other analyses. The simulations of the QI9/HLA complex were 100 ns; the RMSD was calculated from 1,000 frames at 0 ns and for the other analyses from 901 frames at 10 ns. The RMSD per elapsed time was calculated using Equation 1, where N is the number of particles, r*_i_* (t) are the coordinates of the atoms at time t, and r*_i_*^ref^ are the coordinates of the reference structure. The higher the value, the greater the structural change from the reference structure. In this study, the structure at 0 ns was used as the reference structure.

(1) $$\begin{array}{c} RMSD(t)=\sqrt{\frac{1}{N}\sum_{i=1}^{N}{\left(r_{i}(t)-r_{i}^{ref}\right)}^{2}\;} \end{array}$$

The root mean square fluctuation (RMSF) was calculated using Equation 2, where $r_{i}$ are the coordinates of atom *i* and $\langle r_{i}\rangle$ are the mean coordinates of atom *i*. The higher the value, the greater the deviation from the mean position and the greater the fluctuation.

(2) $$\begin{array}{c} RMSF=\sqrt{\langle {\left(r_{i}-\langle r_{i}\rangle \right)}^{2}\rangle }\; \end{array}$$

### Statistics and reproducibility

Data and statistical analysis were performed using Prism 10 (GraphPad Software). For two-way comparison, unpaired Student’s t-test (**Figures 2G**, **5D**, **F, G**; **Supplementary Figure 2B**), unpaired Mann–Whitney t-test (**Figures 3B**, **4C, D**; **Supplementary Figure 2C**), or the paired Wilcoxon signed-rank test (**Figures 3D, E, F**, **4D**) was used.

In **Figures 1B, C**, **Figure 2G**, and **Figure 4D** assays were performed in triplicate. Data are representative of two or three independent experiments.

## Data Availability

The original contributions presented in the study are included in the article/**Supplementary Material**. Further inquiries can be directed to the corresponding author.

## Supplementary material

Supplementary Figure 1 Gating strategy and T cell and cell lines used in this study. **(A)**, Flow cytometry gating strategy of ex vivo tetramer staining in PBMCs from donor GV33V. **(B)**, A24/QI9 tetramer staining of TCR-transduced Jurkat cells. Jurkat cells alone (shaded histogram) or those expressing A24/QI9-specific TCRs (GV33 #7-8, GV33 #1, GV33 #57, GV34 #43, GV34 #34-6, GV36 #11, GV36 #10–2 and GV36 #8C6) (open histogram) were stained with anti-CD3 mAb and A24/QI9 tetramer and then analyzed by flow cytometry. **(C)**, Frequency of SARS-CoV-2 genomes harboring mutations within the QI9 epitope region. The percentage is indicated on the left, and corresponding amino acid variants are shown on the right. Only variants occurring at a frequency of <100 are shown. **(D)**, Flow cytometry gating strategy of tetramer double staining in proliferating T cells from donor GV33 C-2. **(E, F)**, Flow cytometry gating strategy of tetramer double staining in CD8^+^ T cells of splenocytes from mice immunized with pyrQI9 peptide **(E)** splenocytes **(F)**, and lungs **(G)** upon SARS-CoV-2 infection.

Supplementary Figure 2 Frequency and magnitude of T cell response in spleen of peptide-immunized mice in SARS-CoV-2 infection, mRNA expression of inflammatory cytokines in the lung, and structural modeling of pyrQI9/HLA-A24 complex. **(A)**, Representative FACS plots of tetramer double staining in CD8^+^ T cells of splenocytes from mice immunized without peptide (CFA alone, N = 3) or with the QI9 (n=5) and pyrQI9 peptides (n=5) and the frequency of total tetramer^+^ CD8^+^ T cells of splenocytes that encompass QI9/A24, pyrQI9/A24, and both tetramer^+^ populations (cross), and total tetramer^+^ T cells. **(B)**, Magnitude of T cell response of splenocytes to the QI9 or pyrQI9 peptides in mice immunized with CFA alone (n=3), the QI9 (n=5), or the pyrQI9 peptide (n=5). When there was more than one spot in the negative control, positive responses were defined as more than twice that number. The medians are shown with the range. A statistically significant difference between mice is determined by an unpaired Student’s t-test. ns, no statistical significance. *p < 0.05 was considered significant. **(C)**, mRNA expression of inflammatory cytokines such as IFN-γ, TNF-α, CXCL10, and IL-6 in the lung was measured by quantitative PCR, and their gene expression levels were normalized to *Gapdh*. **(D)**, Interaction between QI9 or pyrQI9 and Tyr183 and Arg194 of HLA (Black: QI9, Red: pyrQI9). **(E)**, RMSF of QI9(black) and pyrQI9(red). **(F)**, Distribution of distances between Trp7(Cγ) of QI9 or pyrQI9 and Val176(Cβ) of HLA-A*24:02 molecule.
