# Supplementary material for: N-terminal pyroglutamylation of an HLA-A24-restricted immunodominant epitope enhances SARS-CoV-2-specific T-cell responses
Source: Front Immunol. 2026 Apr 27;17:1758642. doi: 10.3389/fimmu.2026.1758642 (PMC13158186; doi:10.3389/fimmu.2026.1758642)

Supplemental figure 1

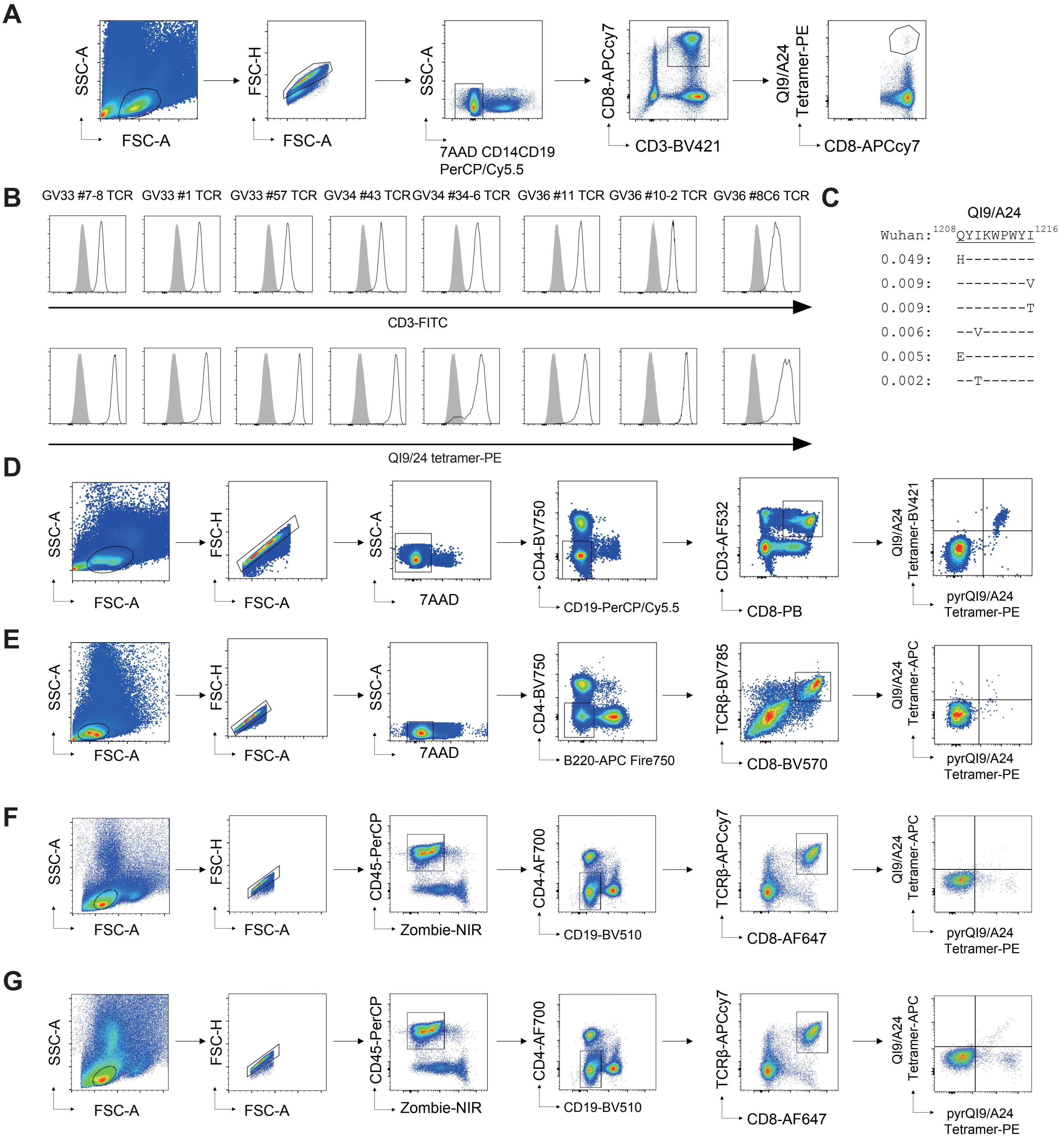

Supplemental figure 2

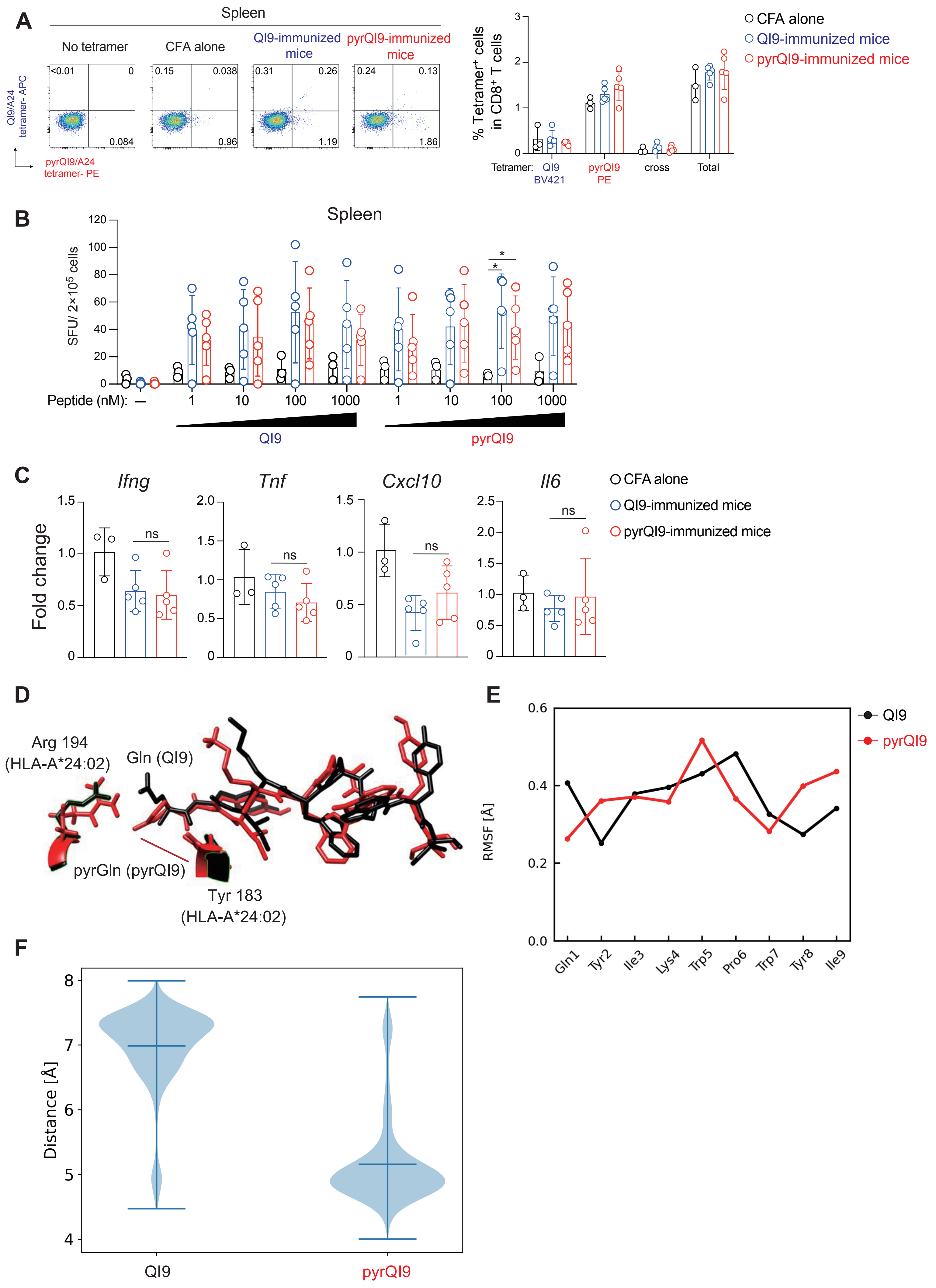

Supplement: Supplementary Figure 1 — Gating strategy and T cell and cell lines used in this study. (A), Flow cytometry gating strategy of ex vivo tetramer staining in PBMCs from donor GV33V. (B), A24/QI9 tetramer staining of TCR-transduced Jurkat cells. Jurkat cells alone (shaded histogram) or those expressing A24/QI9-specific TCRs (GV33 #7-8, GV33 #1, GV33 #57, GV34 #43, GV34 #34-6, GV36 #11, GV36 #10–2 and GV36 #8C6) (open histogram) were stained with anti-CD3 mAb and A24/QI9 tetramer and then analyzed by flow cytometry. (C), Frequency of SARS-CoV-2 genomes harboring mutations within the QI9 epitope region. The percentage is indicated on the left, and corresponding amino acid variants are shown on the right. Only variants occurring at a frequency of <100 are shown. (D), Flow cytometry gating strategy of tetramer double staining in proliferating T cells from donor GV33 C-2. (E, F), Flow cytometry gating strategy of tetramer double staining in CD8+ T cells of splenocytes from mice immunized with pyrQI9 peptide (E) splenocytes (F), and lungs (G) upon SARS-CoV-2 infection. [file DataSheet1.pdf]
